# Design and characterization of *in situ* cell-penetrating multi-modal gadolinium-gold nanoparticles for MR and CT imaging

**DOI:** 10.1016/j.biomaterials.2025.123947

**Published:** 2025-12-24

**Authors:** Alena Kisel, Minrui Luo, Matthew D. Bailey, Kathryn Ghobrial, Luydmila Lukashova, Yiqing Lei, T. Kevin Hitchens, Thomas J. Meade, Michel Modo

**Affiliations:** a Department of Radiology, University of Pittsburgh, Pittsburgh, PA, USA; b Departments of Chemistry, Molecular Biosciences and Neurobiology, Northwestern University, Evanston, IL, USA; c Department of Neuroscience, University of Pittsburgh, Pittsburgh, PA, USA; d Center for Craniofacial Regeneration, University of Pittsburgh, Pittsburgh, PA, USA; e Animal Imaging Center, University of Pittsburgh, Pittsburgh, PA, USA; f Department of Bioengineering, University of Pittsburgh, Pittsburgh, PA, USA

**Keywords:** Gold nanoparticles, Gadolinium, Magnetic resonance imaging, Computer tomography, Contrast agent, DNA, Endocytosis

## Abstract

Contrast agents capable of labeling cells *in situ* are essential for tracking individual cells as they migrate through tissues during dynamic biological processes. Gold nanoparticles (AuNPs) conjugated with gadolinium (Gd) and fluorochromes offer multimodal detection via magnetic resonance imaging (MRI), computed tomography (CT), and fluorescence microscopy. In this study, a systematic strategy was employed to incrementally increase the complexity of Gd-labeled AuNPs (GdAuNPs) and evaluate four distinct surface chemistries for *in situ* cell labeling. Comprehensive characterization of GdAuNP synthesis and stability—using inductively coupled plasma mass spectrometry, UV/visible spectroscopy, transmission electron microscopy, and MR relaxometry—demonstrated high reproducibility and a long shelf-life. Following intracerebroventricular or intrastriatal injection, histological analyses revealed that GdAuNPs labeled over 80 % of neurons in the striatum and approximately 20 % of neural stem cells (NSCs) in the subventricular zone. Only GdAuNPs functionalized with single-stranded DNA (ssDNA) were efficiently internalized by cells; GdAuNPs lacking ssDNA remained extracellular and were removed during immunohistochemical processing. ssDNA-labeled GdAuNPs localized peri-nuclearly following endocytosis. In microglia, GdAuNP also accumulated near the nucleus, whereas in macrophages, all GdAuNP formulations—including those with ssDNA—were mostly sequestered within phagosomes, indicating uptake via phagocytosis. The most effective design, termed type D GdAuNP, featured Gd chelates conjugated both to ssDNA and directly to the AuNP surface. These nanoparticles exhibited the highest MR sensitivity and contrast-to-noise ratio in MRI after *in situ* labeling and were also robustly detected by μCT. This stepwise approach to nanoparticle optimization demonstrates the potential to enhance multimodal imaging sensitivity, supporting the feasibility of a noninvasive visualization of *in situ* labeled neurons and NSCs.

## 1. Introduction

The emergence of noninvasive imaging modalities, such as Magnetic Resonance Imaging (MRI) and Computed Tomography (CT), has revolutionized the field of medical diagnostics, offering an exquisite resolution and a detailed visualization of deeply seated anatomical structures in living subjects [1]. However, these imaging modalities currently predominately report on tissue properties (e.g., water density or diffusion), rather than the identity or phenotype of constituent cells. To visualize cells using MRI, the incorporation of contrast material into individual cells is required [2]. However, most tissue-based cells do not readily incorporate MRI contrast agents. Cells of the reticuloendothelial system (RES) phagocytose nanoparticle contrast agents, such as ultrasmall particles of iron oxide (USPIO), to afford their MR imaging as part of the clearing process [3,4]. Recent evidence also suggests that there is some uptake of monomeric gadolinium-based contrast agents (GBCA) in the blood by circulating cells [5], with some long-term retention in brain tissue [6]. However, these non-phagocytotic routes to cellular uptake are commonly minute and typically well below the MRI detection limit [7].

For MRI applications, high-sensitivity agents based on nanoparticle platforms are appealing for cellular tracking. Most nanoparticle-based MRI contrast agents have a spherical morphology, with cellular uptake being significantly influenced by hydrodynamic diameter [8]. Particles with diameters between 0.5 and 10 μm are known to be susceptible to phagocytosis by RES cells. In contrast to phagocytosis, endocytic processes include macro-pinocytosis (0.1–5 μm size particles), or clathrin- (~120 nm) and caveolin-based pinocytosis (~80 nm). Passive uptake via direct membrane penetration (<10 nm particles) is also possible and heavily influenced by overall nanoparticle surface charge [8–10]. Smaller particles, notably nanoparticles, are therefore candidates for labeling non-phagocytic cells [8,11]. However, these uptake processes are typically inefficient and can result in an accumulation of the particles within endocytic vesicles that eventually undergo exocytosis [12]. Sequestration of contrast agent in lysosomal compartments can also quench their relaxation properties by restricting access to free water, as in the case T_1_-weighted contrast of GBCA [13,14]. *In vivo* labeling of non-phagocytic cells, such as those of the neuronal cell lineage, with contrast agents is therefore challenging.

There is an extensive literature on *in vitro* uptake of contrast agents into cells for MRI detection to monitor their distribution and migration after tissue implantation [15–17]. However, very few studies have addressed *in situ* cellular uptake of contrast agents after intracerebroventricular (ICV) or intraparenchymal injections [18,19]. MR imaging of individual cells within a tissue requires high relaxivity agents that provide sufficient contrast compared to the tissue background, but also an efficient uptake mechanism and retention within the targeted cells [2,20]. Micron-sized particles of iron oxide (MPIOs) provide a high T2 relaxivity that affords the MR detection of even a single particle [21], but these agents cause a major dephasing of neighboring voxels (due to superparamagnetism). Determination of the precise tissue location of a cell is hence challenging. In the case of many MPIO-labeled cells, a significant signal loss occurs, precluding the definition of anatomical context around the labeled cells. Signal loss (i.e., hypointensity) on T_2_-weighted MR images can also be caused by air bubbles and small bleeds, which can occur due to needle injections of contrast material or cells, leading to an ambiguity in image interpretation. Signal gain (i.e., hyperintensity) on T_1_-weighted (T1w) MR images therefore provides a more unequivocal MRI signal, as signal gain on T1w images is a less common artifact.

The ability to trace the location of neuronal cells and monitor the migration of endogenous neural stem cells (NSCs) within developing, repairing, or regenerating brain tissue could significantly enhance our understanding of these biological processes [20,22,23]. T1w MR imaging, combined with contrast agents capable of *in situ* cell labeling and long-term intracellular retention, offers several unique advantages for this purpose. To meet these requirements, gadolinium-based contrast agents (GBCAs) must exhibit high cellular uptake in non-phagocytic cells, such as NSCs. Moreover, the agents should become compartmentalized within the cells to ensure long-term retention. Ideally, the contrast agent should also include a fluorescent moiety to allow orthogonal confirmation of cellular uptake and localization via histological analysis [2,17].

Gold nanoparticle (AuNP)-based platforms offer a promising solution for simultaneously achieving high MR sensitivity, effective cellular uptake and retention, as well as compatibility with multimodal imaging [24,25]. The ease of functionalizing AuNPs through strong Au–S bonding with diverse chemical species makes them highly versatile in biomedical applications [26]. Additionally, the AuNP core serves as a contrast agent for computed tomography (CT) [27,28]. Conjugating GBCAs into multimeric complexes on the AuNP surface (GdAuNPs) markedly enhances relaxivity and sensitivity for MR imaging [24–30]. Incorporating fluorescent moieties, such as cyanine dyes, further enables multimodal tracking by MRI, CT, and fluorescence. Previously, we functionalized GdAuNPs with polyT single-stranded DNA (ssDNA), yielding DNA@GdAuNPs with high nanoparticle stability and the ability to detect transplanted NSCs *in vivo* using T1w MRI [16,29].

In this study, we present a systematic framework for the design of Gd-functionalized AuNPs and evaluate their effects on MRI relaxivity and cellular uptake during *in situ* labeling. We describe four GdAuNP formulations—Types A, B, C, and D—each with increasing design complexity.

- **Type A:** AuNPs with a monolayer of adsorbed Gd complexes.
- **Type B:** AuNPs with both adsorbed Gd complexes and polyT ssDNA.
- **Type C:** Gd complexes covalently linked to polyT ssDNA, without surface modification of the AuNP core.
- **Type D:** A combination of Types A and C, with Gd complexes bound both to the AuNP surface and to ssDNA.

We conducted a comprehensive physicochemical characterization of these formulations and assessed their ability to label neural cells *in situ*. Our objective was to identify the GdAuNP design most suitable for cell tracking using MRI or micro-CT (μCT). This study aimed to determine cell-penetration of GdAuNP into brain cells after intracerebral delivery. Two main microenvironments were compared, notably (1) intracerebroventricular injections, which resulted in GdAuNP in an aqueous environment with uptake into mitotic neural stem cells/progenitor cells in the subventricular zone (SVZ), and (2) intraparenchymal striatal injections, consisting of a post-mitotic neuronal environment. The impact of the microenvironment on GdAuNP cellular uptake and contrast enhancement were evaluated in the rat brain using MRI and μCT. Additionally, fluorescence microscopy was used to determine which GdAuNP type was most efficiently internalized by proliferating NSCs and post-mitotic neurons. This systematic approach to GdAuNP design and evaluation offers a versatile platform for imaging both transplanted and endogenously labeled neural cells. It enables detailed investigations of cellular contributions to brain architecture and function via MRI and CT.

## 2. Methods

### 2.1. GdAuNP design and production

#### 2.1.1. General experimental methods

Reagents were purchased of reagent grade purity or higher and used without further purification unless otherwise noted. Ultrapure water (Type I, resistivity ≥18.2 MΩ cm) was used for all aqueous solutions. Gd-609 and Gd-828 were synthesized using previously reported methods [29]. All glassware to be used with AuNPs was washed with *aqua regia*, rinsed thoroughly with water, and dried prior to use. 15T – single strand DNA (ssDNA) for type B particles were purchased from Integrated DNA Technologies, and amino modifier C6 dT, 3′-Thiol-Modifier 6 S–S, and Cy3 phosphoramidite for synthesizing 24T-Gd-ssDNA (3’ – S–S TTT-TTT-TTT-T*TT-T*TT-T*TT-T*TT-T*TT-Cy3 – 5′) were purchased from Glen Research.

#### 2.1.2. High performance liquid chromatography

Analytical HPLC was carried out with an Agilent 1260 Infinity II LC system equipped with a 1260 Infinity II Qua-ternary Pump, an inline diode array UV–Vis detector and Agilent 6120 Quadrupole LCMS System. DNA elution was monitored at 260 nm. The column used was a XBridge C18 5 μm reversephase column (4.6 × 150 mm) and a DNA-Pac^™^ PA200 Analytical column (4.0 mm × 250 mm, 8 μM, ThermoFisher).

Semi-preparative HPLC was carried out with an Agilent Prepstar system equipped with two PrepStar 218 solvent delivery modules, an inline diode array UV–Vis detector, and 440-LC Fraction Collector. Absorbances at 260 nm were monitored. The column used for semi-preparative scale HPLC was an XBridge Prep C18 5 μm OBDTM reverse phase column (19 × 150 mm).

#### 2.1.3. Matrix-assisted later desorption/ionization time of flight measurements

To prepare MALDI samples, a matrix was created by dissolving 2,5-dihydroxyacetophenone (DHAP, 25 mg) in methanol (333 μL). Samples of oligonucleotide solutions (0.5 μL) combined with MALDI matrix (1 μL) were spotted on 10 spot coin chips and left to dry in air. Coin chips were placed in an MTP MultiProbe Adaptor for analysis. MSI was performed in negative ion linear mode over a mass range of *m*/*z* 4000–20000 on a rapifleX MALDI Tissuetyper TOF mass spectrometer (Bruker) at 80 % laser power.

#### 2.1.4. DNA synthesis

24T-Gd-ssDNA oligonucleotides (*Product 1*) were synthesized on solid-phase-controlled pore glass beads (CPGs) by standard techniques on a MerMade automated synthesizer. Both dT and amino modifier C6 dT (indicated as T*) were incorporated onto 3′-Thiol-Modifier 6 S–S controlled pore glass beads, along with Cy3 phosphoramidite (Supplementary Figure 1). To remove *Product 1* from the resin, it was subjected to 30 % ammonium hydroxide treatment overnight, with subsequent removal of the ammonium hydroxide using nitrogen. The strands were isolated from CPG beads through five filtration cycles. *Product 1* was collected and purified with a Xbridge column on a semi-preparative scale HPLC with solvent A = 0.1 Triethlyamine Acetate (TEAA) buffer in water and solvent B = acetonitrile. The elution followed (t = 0 min, 5 % B; t = 5 min, 5 % B; t = 50 min, 50 % B; t = 51 min, 80 % B; t = 56 min, 80 % B; t = 57 min, 5 % B). *Product 1* was followed by deprotection of Dimethoxytrityl (DMT) with 3 mL of 20 % acetic acid, shielded with aluminum foil, and rotated for 2 h to get *Product 2*. This was extracted three times with ethyl acetate by centrifuging at 3000 rcf for 5 min and characterized by MALDI-TOF-MS. *m*/*z*: [M – H]^−^ Calcd for C_319_H_415_N_60_O_178_P_24_S_2_ 8745.57; Found 8745.66.

The peptide coupling of an azide on the ssDNA was accomplished by dissolving 400 nmol of DNA in 500 μL of 0.1 M sodium bicarbonate buffer at pH 9. Subsequently, 6 mg of azidobutyrate NHS ester from Glen Research, dissolved in 100 μL of acetonitrile, was added to the DNA-buffered solution and rotated at room temperature for 3 h before desalting with a NAP-25 Sephadex column. The crude ssDNA (*Product 3*) was characterized using MALDI-TOF-MS. *m*/*z*: [M – H]^−^ Calcd for C_339_H_438_N_75_O_183_P_24_S_2_ 9299.08; Found 9299.35.

The copper click reaction between Gd-609 and DNA was carried out under nitrogen. It involved dissolving 200 nmol DNA in 500 μL of 2.0 M TEAA buffer with 1 μmol of copper sulfate, 5 μmole of tris-hydroxypropyl triazolyl amine, 100 μmole of Gd-609, and 10 μmole of sodium ascorbate. The reaction was rotated for 2 days, and the yielded *Product 4* was characterized by MALDI-TOF-MS. *m*/*z*: [M – H]^−^ Calcd for C_439_H_587_N_100_O_218_P_24_S_2_ 12342.74; Found 12342.66.

Final separation was accomplished using a DNAPac^™^ PA200 Analytical column (4.0 mm × 250 mm, 8 μM, ThermoFisher). Mobile phases consisted of A: tris buffer (pH 8.0), and B: tris buffer containing 1.25 M NaCl (pH 8.0). The elution followed (t = 0 min, 32 % B; t = 17 min, 46.4 % B; t = 17.1 min, 75 % B; t = 19.1 min, 75 % B; t = 19.2 min, 32 % B; t = 24 min, 32 % B). Fractions were collected at 3–5 min.

#### 2.1.5. GdAuNP synthesis

AuNPs (13 nm) were synthesized using the Frens method. HAuCl_4_ trihydrate (39.4 mg) was dissolved in water (99 mL). The solution was heated to reflux. A solution of sodium citrate (114.1 mg) was dissolved in water (1 mL) and added to the reaction. The solution's color underwent rapid transformation to colorless, black, and ultimately to wine-red. After 15 min, the reaction was removed from the heat and filtered through a glass frit funnel. The particles were then characterized using UV–vis spectroscopy, ICP-MS, and TEM imaging.

Lyophilized ssDNA (200 nmol) underwent deprotection using 600 μL of 100 mM dithiothreitol (DTT) in phosphate buffer (180 mM, pH 8.0). The solution was rotated at room temperature for 2 h and passed through a pre-packed G25 Sephadex column (NAP-25, GE Life Sciences) using 180 mM phosphate buffer as the mobile phase. ssDNA collection was monitored visually by observing the Cy3 dye on the DNA.

**Fig. 1.**
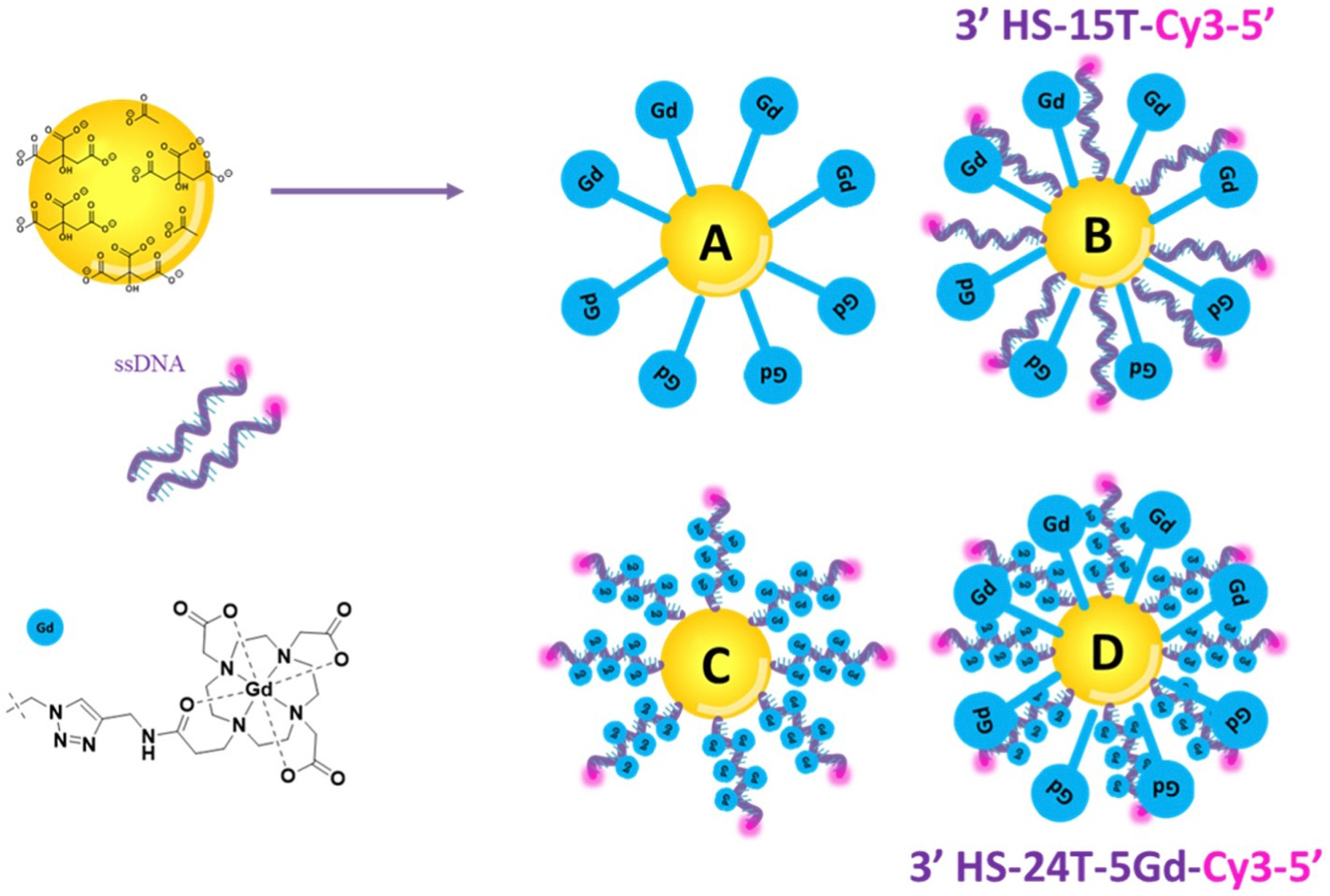
Conceptualization of design characteristics of gadolinium-gold nanoparticles (GdAuNP). To produce paramagnetic MRI-detectable gold nanoparticles (AuNP), gadolinium chelates can be conjugated to AuNP. In its simplest design configuration, these are directly attached to the AuNP, which we termed Type A GdAuNP. Fluorescent moieties can be further attached to afford detection of these GdAuNP by fluorescent microscopy. To improve cell uptake and retention of these, conjugation of single stranded DNA (ssDNA) (3′–HS–TTT-TTT-TTT-TTT-TTT-Cy3–5′) can be added to these GdAuNP to create Type B designs. To increase Gd loading and improve their water exchange characteristics, Gd chelates can be conjugated to the ssDNA strands to create Type C GdAuNP with ssDNA 3′–HS–9T-T*TT-T*TT-T*TT-T*TT-T*TT-Cy3–5’. Type D GdAuNP further enhances Gd loading by backfilling Type C GdAuNP with additional Gd chelates directly conjugated onto the AuNP (as in the Type B design). (For interpretation of the references to color in this figure legend, the reader is referred to the Web version of this article.)

All ligand exchange reactions were done in the presence of 0.01 % Tween 20. To introduce gadolinium functionality to **Type A** AuNPs (Fig. 1), 2000 equivalents per AuNP Gd-828 were added in methanol (500 μL). The particle mixture was rotated at room temperature to minimize the likelihood of Gd collision with AuNPs, thereby increasing stability and Gd loading on Type A particles.

**Type B** particles involved the salt aging of 15T-ssDNA onto AuNPs. Specifically, 100 equivalents of ssDNA were added to 75 mL of AuNPs in the presence of Tween 20. The solution was sonicated for 30 s and stirred for 30 min. Over the subsequent 5 h, a solution of 4.7 M sodium chloride, phosphate buffer, and 0.01 % Tween 20 was added incrementally with 630 μL, 645 μL, 657.5 μL, 672.5 μL, and 690 μL each time, with each addition followed by 30 s of sonication. The final NaCl concentration reached 600 mM, and the solution was rotated overnight. After purification with DPBS, 2000 equivalents of Gd-828 were dissolved in Millipore water and added to the DNA-conjugated AuNPs using the same functionalization procedure as for Type A particles.

**Type C** particles utilized Gd-coupled 24T-Gd-ssDNA, following the same conjugation procedure as Type B particles but without direct Gd conjugation.

**Type D** particles involved direct Gd-828 conjugation, akin to Type B particles.

The GdAuNPs were purified via a series of three centrifugation cycles (each lasting 50 min at 20,000 rcf), followed by resuspension, continued until no discernible dye was visible in the supernatant. The resulting purified and functionalized GdAuNPs underwent sterilization using a 0.2 μm PES filter and were subsequently concentrated with a spin filter at a centrifuge speed of 1500 rcf for 5 min. Subsequent analyses encompassed UV–vis and TEM for particle size determination, ICP-OES for assessing particle concentration and loading through examination of the Gd/Au ratio, as well as relaxivity measurements conducted at both 1.41 T and 9.4 T.

### 2.2. GdAuNP physicochemical characterization and quality assurance

#### 2.2.1. Ultraviolet–visible spectroscopy

The UV–Vis spectra of citrate-stabilized gold nanoparticles (AuNPs) and gadolinium-functionalized AuNPs were acquired using a Cary 5000 spectrophotometer in a cuvette with a 1 cm path length. Concurrently, single-strand DNA analysis was performed using a nanodrop cell with a 1 mm path length. The obtained spectra revealed a consistent alignment of citrate-stabilized particles, indicating uniform sizes across all six batches of AuNPs. The nanoparticle sizes were determined using the formula $d=exp\left(B_{1}\frac{A_{spr}}{A_{450}}-B_{2}\right)$; with the constants *B*_1_ = 3.00, *B*_2_ = 2.20 [30].

#### 2.2.2. Transmission electron microscopy

For the preparation of samples intended for transmission electron microscopy (TEM), copper TEM grids coated with a carbon layer were selected as the support film. Given the inherent hydrophobic nature of these grids, ensuring a uniform distribution and adhesion of aqueous particle solutions presented a challenge. To address this, a 30-s glow discharge treatment was performed using the Pelco EasiGlow Glow Discharge system, effectively inducing a charge on the grids to impart hydrophilicity. Subsequently, a diluted solution containing Type A, B, C, and D AuNP samples (approximately 5 μL) was carefully applied to the grid surface, followed by the removal of excess solution using Whatman filter paper. After a brief 1–2 min drying period to facilitate particle settling, the sample was ready for analysis. For imaging purposes, TEM micrographs were acquired using the Hitachi HD-2300 Dual EDX Cryo STEM operating at a voltage of 200 kV at varying magnifications. The local elemental mapping and elemental compositions were determined by energy-dispersive X-ray spectrometry (EDX) with dual-EDX system under the TE mode with 148 frame scans and processed with kernel size 3.

Subsequent analysis was conducted with ImageJ following the conversion of FFT/bandpass filter from 3 to 40 and a threshold was applied, as well as the watershed. Particles were analyzed excluding circularity below 0.5 and size greater than 3 pixels. Particle areas were then collected from ImageJ, confirmed for overlap with raw images, and converted to diameters.

#### 2.2.3. Inductively couple precipitation mass spectrometry

The analysis of AuNPs samples was conducted via ICP-OES using the Thermo iCAP 7600 instrument. For samples containing a combination of gold, gadolinium, and phosphorus components, a digestion process was carried out. To prepare the samples, a digestion procedure was performed using a 1:1 mixture of hydrochloric acid (37 %, 200 μL) and nitric acid (70 %, 200 μL). Subsequently, the mixture was heated to 65 °C for a duration of 3 h. Following this, the acidic matrix was adequately diluted with Millipore water to attain a total acid concentration of 4 %. The concentrations obtained were calculated considering the applied dilution factor. In contrast, all the materials involved in the needle experiments were analyzed using ICP-MS (Thermo iCAP Q), which encompasses Au, Gd, and Fe elements.

#### 2.2.4. Dynamic light scattering

The hydrodynamic diameter and size distribution of GdAuNPs were measured using a Malvern Zetasizer Ultra instrument equipped with a 4 mW, 633 nm He–Ne laser. The detection angle for backscatter measurements was set at 173°. AuNP samples were diluted to a concentration of approximately 10 nM and 100 nM in DPBS and analyzed using a disposable micro cuvette (Malvern Panalytical ZEN0040) at position of 3 mm. The reported values are an average of 10 consecutive measurements for both the citrate-stabilized AuNPs and the functionalized AuNPs. The temperature was set to 25 °C and allowed to equilibrate for 30 s. The attenuator was set to 7 for citrate-stabilized AuNPs; it was adjusted to 8 for type A, C and D AuNPs and further adjusted to 9 for Type B AuNP to obtain a mean count rate around 300 kcps. Data was processed using ZS XPLORER software to determine size distributions. The refractive index used for the analysis of AuNPs is 0.18, and the absorption extinction coefficient is 3.433. The refractive index for the solvent is 1.33, and its viscosity is 0.8872.

Zeta potential of each type of AuNP were measured with Malvern Zetasizer Ultra and disposable folded closed capillary zeta cell (Malvern Panalytical DTS1070). AuNPs were diluted to approximately 10 nM with ultrapure H_2_O and 1 mL of sample were transferred to the capillary cell using a 1 mL disposable syringe. The attenuation, voltage and number of runs were set to automatic. The samples were kept at room temperature and equilibrated at 25 °C for 30 s. Three consecutive runs were taken for each type of AuNPs, with 60 s pause between each repeat. The measurement position were 2 mm, the detector angle was 17°, the applied voltage was 150 V, and the attenuator was 10 for Type C AuNPs and 11 for all other types.

### 2.3. MR relaxometry

#### 2.3.1. Nanoparticles relaxivity in 1.41 T (Northwestern)

The relaxivity of GdAuNPs was measured by analyzing the relationship between 1/T1 values at 1.41 T and the corresponding Gd concentration, determined via ICP-OES. An arbitrary dilution series was made for each sample. All sample solutions were equilibrated to a temperature of 37 °C for thermal stability prior to T1 relaxation time measurements. T1 relaxation times were measured on a Bruker Minispec mq60 NMR spectrometer (1.41 T, 60 MHz) using an inversion recovery pulse sequence with a repetition time (TR) of 5000 ms, and 7 data points were collected. The longitudinal relaxation rate (1/T1, s^−1^) was plotted against Gd concentration (mM) for each dilution. The slope of the resulting linear fit was taken as the relaxivity (r1, mM^−1^s^−1^). The linear fit of the data was confirmed by a coefficient of determination (R^2^) value greater than 0.99 for each experiment. The final relaxivity values were averaged from multiplicate experiments (n = 3) to provide reliable estimates of the magnetic property characterization.

#### 2.3.2. Nanoparticles relaxivity in solution (Northwestern)

High field relaxivity measurements were performed on a 9.4T 30-cm Bruker Biospec MRI System equipped with a B-GA12 gradient set, running Paravision 6.0.1. (Bruker Biospin Inc, Billerica, MA, USA). Stocks of GdAuNP conjugates were serially diluted to the following concentrations of nanoparticles: 500 nM, 250 nM, 100 nM, 50 nM, 25 nM, 10 nM, 5 nM, 2.5 nM, 1 nM. Dulbecco phosphate buffered saline (DPBS) was used as a baseline for comparison. Samples were flame-sealed in 1 mm diameter glass capillary tubes, secured in a larger tube filled with water and imaged using a 23 mm diameter birdcage RF coil. T1 was measured using a variable repetition time accelerated spin-echo sequence (RARE-VTR) with the following parameters: Variable TR = 147, 200, 400, 800, 1500, 3000, 6000, and 10,000 ms; TE = 6.8 ms, field of view = 2 cm × 2 cm, matrix size = 200 × 200, resolution = 100 μm, number of axial slices = 3, slice thickness = 1 mm, and 2 averages. T1 was fit monoexponentially using regions of interest drawn around each sample. Nanoparticle relaxivity (r1) was computed by linear regression of the relaxation rates (R1 = 1/T_1_, in s^−1^) vs. the nanoparticle (in nM) and Gd (in mM) concentrations.

#### 2.3.3. Nanoparticles relaxivity in solution (Pittsburgh)

To ensure stability of MR properties of nanoparticles, their relaxivity was measured again within 2 days after arrival. The same capillaries were placed in a Bruker 9.4 T 30 cm magnet with a B-GA12 HD gradient set. For R1 (1/T_1_) measurements, a fast spin echo sequence with variable TR was used, whereas a multi-slice multi-echo (MSME) sequence was chosen for R2 (1/T_2_) measurements (for details of all imaging acquisition parameters see Table 1). A linear regression was performed to determine the relaxivities (r_1_ and r_2_), signal-to-noise ratio (SNR), contrast-to-noise ratio (CNR), and T_1_w contrast of agents.

### 2.4. microCT of GdAuNP in solution

Sets of glass capillaries with contrasts agent were used for microcomputed tomography (micro-CT) analysis on Scanco μCT 50 (Scanco Medical, Brüttisellen, Switzerland) system. 20 μm and 6 μm voxel sizes, 55 kVp, 145 μA intensity, 0.36° rotation step (180° angular range) and 1000 ms exposure per view were used for the scans. The Scanco μCT software OpenVMS (HP, DECwindows Motif 1.6) was used for 3D reconstruction and viewing images. Total Volume (TV), Particles Volume (PV) and Particles Volume Fraction (PV/TV representing particles concentration) were calculated.

### 2.5. Reactivity of GdAuNP with injection needle

As GdAuNP are injected through a ferrous injection needle, potential interactions between these metals could occur. To determine if ejection leads to changes in Gd, Au and Fe content in the ejectate, nanoparticles (500 nM) were suspended and diluted in dH_2_O prior to loading these through needles (32G, 7803–04, Hamilton) into 50 μL glass syringes (7637–01, Hamilton). Manual ejection occurred straight after loading. Experimental conditions (n = 3/condition) consisted of 1) an unused syringe/needle without any GdAuNP (0 pass); 2) a single GdAuNP ejection (1 pass), 3) 10x GdAuNP ejections with cleaning after each pass (10 passes). After each pass of GdAuNP (35 μL), the syringe/needle were cleaned 3 times with cleaning solution (18311, Hamilton Company) followed by deionized water 3 times (Supplementary Figure 2). All ejected liquids were collected for analyses.

Ejectates were collected and acid-digested in 400 μL of a 1:1 mixture of concentrated hydrochloric acid (37 %) and nitric acid (70 %). Entire needles were also digested in 1 mL of the same acid mixture. All samples were incubated at 65 °C for 72 h to ensure complete digestion of gold nanoparticles and metal components. Final digests were diluted with Milli-Q water to reach a total acid concentration of 4 % prior to analysis. Gadolinium (Gd), gold (Au), and iron (Fe) concentrations were quantified for both the needle digests and ejected solutions using ICP-MS (Thermo iCAP Q) and reported in ng/g.

### 2.6. Animal experiments

All animal procedures complied with the US Animals Welfare Act (2010) and were approved by the University of Pittsburgh Institutional Animal Care and Use Committee (IACUC). Sprague-Dawley rats (male, n = 5/condition, N = 20, 280 ± 15 g, Taconic Bioscience, USA) were maintained on a 12-h light/dark schedule, with food and water available *ad libitum.*

### 2.7. Impact of injection environment on GdAuNP contrast

To compare contrast provided by GdAuNP in different tissue microenvironments, nanoparticles were injected into the right lateral ventricle (liquid) and right striatum (tissue) of healthy rats. Nanoparticle solutions were injected under isoflurane anesthesia (4 % induction, 1 % maintenance in 30 % O_2_) using a frame-mounted injection pump (World Precision Instruments, USA) through a 50 μL Hamilton syringe with a 32 G beveled stainless-steel needle (Hamilton, USA). 30 μL of GdAuNP solution was injected into the right lateral ventricle and 4 μL of GdAuNP solution was injected into the right striatum. The left hemisphere served as a control condition for measurements. Stereotaxic coordinates were determined in accordance with the rat brain atlas [31]: 1) the lateral ventricle was targeted at anterior-posterior −0.5 mm from bregma, medial-lateral −1.5 mm from the middle line, and dorsal-ventral −4.0 mm from the brain surface; 2) the striatum was targeted at anterior-posterior −0.5 mm from bregma, medial-lateral −3.5 mm from the middle line, dorsal-ventral −4.5 mm from the brain surface. The injection rate was 10 μL/min for the intraventricular injection and 1 μL/min for the striatal injection. After injection, the needle was left in place for 10 min before being slowly removed to minimize liquid backflow. Bone wax was used to fill the burr holes from the stereotactic injections. Lidocaine topical cream (4 %, Generic) was applied as an analgesic, as well as systemic buprenorphine (0.05 mg/kg i.p., every 12 h) to provide sustained pain relief after suturing of the skin incision.

### 2.8. Terminal procedures

Animals were euthanized using transcardial perfusion 24 h post-injection. After terminal anesthesia (1 mL/kg, Fatal Plus, Covetrus), blood was first flushed transcardially with phosphate buffered saline (PBS), and then tissue was fixed by transcardial perfusion with 4 % paraformaldehyde.

### 2.9. High resolution 3D MR imaging of GdAuNP in ventricles and tissue

High-resolution 3D MRI was performed on a 9.4 T Bruker scanner. T_1_w and T_2_w images were acquired with 0.1 × 0.1 × 0.1 mm and 0.2 × 0.2 × 0.2 mm resolution, respectively. Acquisition parameters are presented in Table 1. Slices in a coronal orientation served as a reference for μCT and histology. Coronal slices were used to measure GdAuNP contrast.

### 2.10. μCT of GdAuNP after implantation

Rat brains were used for microcomputed tomography (μCT) analysis on a Scanco μCT 50 (Scanco Medical, Brüttisellen, Switzerland) system using a 20 μm voxel size, 55 kVp, 145 μA intensity, 0.36° rotation step (180° angular range) and 1000 ms exposure per view. The Scanco μCT software OpenVMS (HP, DECwindows Motif 1.6) was used for 3D reconstruction and quantification of images.

### 2.11. Histological analyses

After removal from the skull, brains were post-fixed in 4 % paraformaldehyde for 24 h prior to being cryopreserved in 30 % sucrose with sodium azide (Sigma) at 4 °C. Histological sections (50 μm thickness) were cut on a cryostat (Leica) directly onto microscopic slides to preserve tissue morphology and avoid washing out of GdAuNP. Brain sections were incubated in blocking buffer (5 % BSA in TBS: PBS + 0.1 % Triton X-100 (Sigma)) at room temperature (21°C), washed 3 times with 0.01 M PBS, followed by overnight incubation at room temperature with primary antibodies (Table 2). After 18 h, primary antibodies were washed off (3x PBS), and appropriate secondary AlexaFluor 488 or 647 antibodies (1:1000, Invitrogen) were applied for 1 h at room temperature. Antibodies were removed with 2x PBS washes and one with distilled water. Nuclear counterstaining was achieved with the nuclear marker Hoechst 33342 (1 μg/mL, Sigma). After 3x washes, sections were coverslipped with Vectashield for fluorescence and stored at 4 °C prior to imaging. Visualization of antibodies was performed with a fluorescence microscope (Axioimager M2, Zeiss) interfaced with a monochrome camera driven by Zen Blue software (Zeiss) using a motorized stage.

### 2.12. Histology quantifications

Cells labeled with GdAuNP were counted in the subventricular zone (SVZ) of the right lateral ventricle and in the striatum (Supplementary Fig. 3) in several regions of interest (ROIs). Cells in the SVZ were counted in >5 ROIs (600 × 600 μm). In the striatum, two groups of ROIs were placed: central ROIs (n > 4, min size 600 × 600 μm), as close to needle track and injection site as possible, and peripheral ROIs (n > 5, min size 600 × 600 μm), approximately 3–5 mm away from the injection site. Cells were counted in Fiji/ImageJ manually.

### 2.13. Scanning electron microscopy & energy-dispersive X-ray spectroscopy

Rat brain slices (50 μm thickness) were prepared for scanning electron microscopy (SEM) and elemental analysis following nanoparticle injection and fixation. Slices were mounted on aluminum stubs and coated with a thin layer of carbon using a carbon evaporator to minimize charging during imaging. SEM was performed using a FEI Quanta 650 Environmental SEM (ESEM) operated in high-vacuum mode. Elemental composition was assessed via energy-dispersive X-ray spectroscopy (EDX) to detect and map gold (Au) content in the tissue sections. EDX spectra and elemental mapping were collected using an Oxford Instruments X-Max detector and analyzed with Oxford AZtecEnergy software. Images and EDX maps were acquired from multiple regions of interest to evaluate the distribution and presence of AuNP within brain tissue.

### 2.14. Statistical analyses

Graphing and statistical analysis of data was performed using Prism 10 (GraphPad) with data points representing the mean and bars reflecting the standard deviation. Statistical significance was set at p < 0.05. The acquired data was of a parametric nature and afforded comparisons using a one-way analysis of variance (ANOVA) followed by Bonferroni's post-hoc test. A Pearson correlation was computed to determine the strength of association between two measurements (e.g. MRI and μCT).

## 3. Results

### 3.1. Design, synthesis and physicochemical characterization of GdAuNP

To enable cellular MRI using a modular nanoparticle platform, 4 types of gadolinium-functionalized gold nanoparticles (GdAuNPs) were designed, each incorporating different surface configurations to enhance MR contrast, as well as cellular uptake and retention. The core design of GdAuNP (i.e. Type A) involves gadolinium chelates directly conjugated to citrate-stabilized gold nanoparticles (Fig. 1). This minimal construct was progressively elaborated to incorporate fluorescently labeled single-stranded DNA (ssDNA) for corroborative histological imaging (Type B) of improved cellular uptake/retention. DNA-conjugated Gd chelates further enhanced relaxivity and retention *in vivo* (Type C). Finally, a Type D design combined the DNA-conjugated Gd (Type C design characteristics) with additional Gd directly attached to the AuNP surface (Type B design characteristics) through backfilling. Type D hence maximized cellular uptake/retention, as well as Gd loading for MR imaging. Au and Gd also convey this contrast agent the potential to be detected by both MRI and μCT, whereas the fluorescence moiety on the ssDNA strands affords detection using fluorescence microscopy (i.e. tri-modal detection).

The synthetic procedures for Gd chelates and ssDNA constructs (Supplementary Fig. 1) were characterized using HPLC-MS for Gd-609 (Supplementary Figure 4A), Gd-828 (Supplementary Figure 4B), as well as ssDNA (Supplementary Figure 4C) to confirm identity and purity. MALDI-TOF mass spectrometry validated the successive conjugation steps, with precise agreement between calculated and observed masses for the deprotected strand (8745.57 Da) (Supplementary Fig. 5A), azide-coupled intermediate (9299.08 Da) (Supplementary Fig. 5B), and the final Gd-609-labeled product (12,342.74 Da) (Supplementary Fig. 5C) of 3′ S–S-TTT-TTT-TTT-T*TT-T*TT-T*TT-T*TT-T*TT–Cy3 5’. The sharp and well-resolved peaks, with expected mass/charge ratios, confirmed successful synthesis. The measurement of the Gd-labeled ssDNA constructs matched with a high accuracy the calculated molecular weights.

**Fig. 2.**
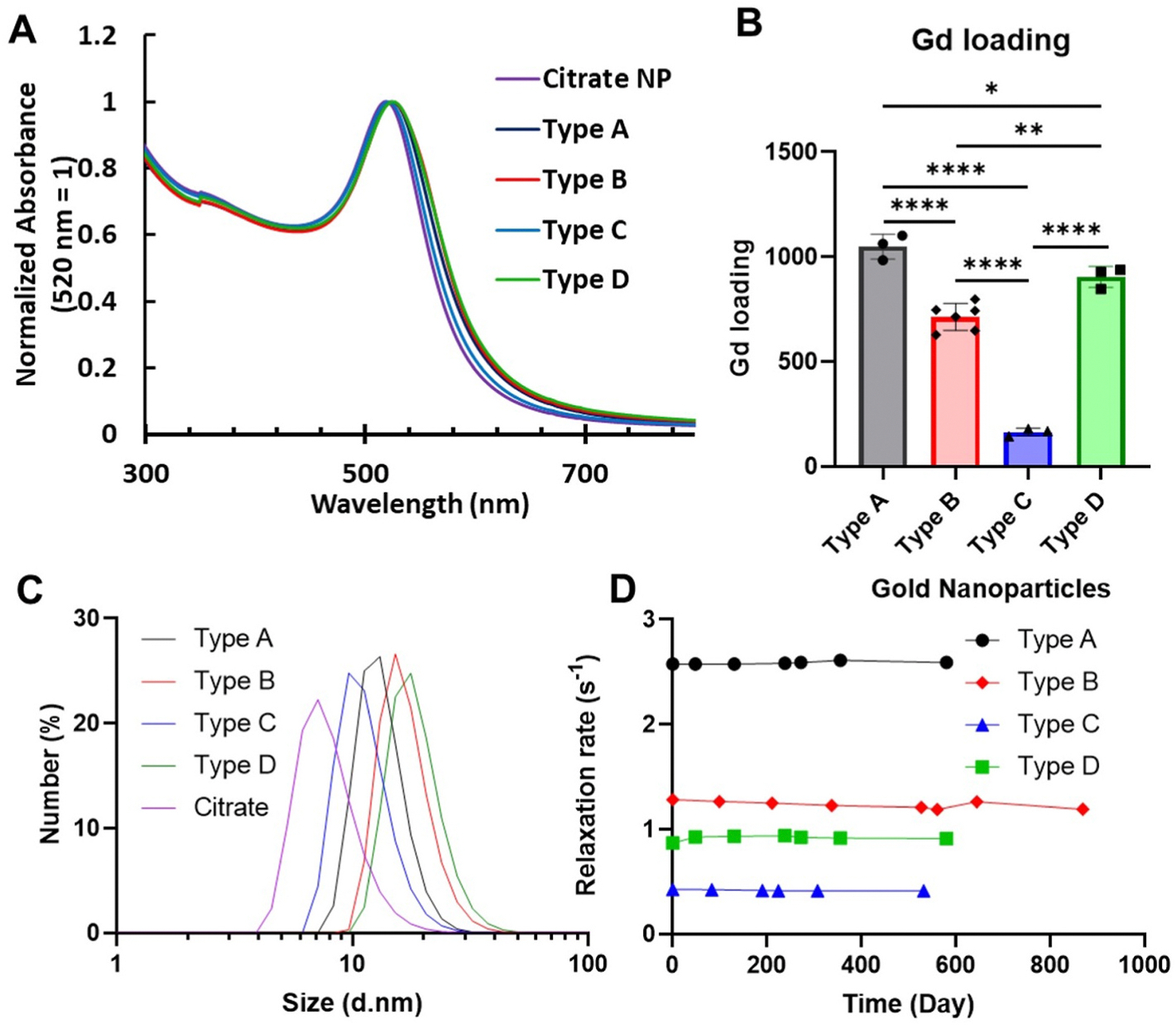
Size, shape, structure and chemical properties of Gd-DNA-AuNPs. **A**. UV–vis spectrum for citrate stabilized AuNPs and functionalized Type A, B, C, D Gd-DNA-AuNPs, normalized at 520 nm wavelength. **B**. Gd loading for functionalized Type A, B, C, D GdAuNP (n = 3) achieved with ICP-MS. (*p < 0.05; **p < 0.01, ***p < 0.001, ****p < 0.0001) **C**. Size distribution by number of citrate stabilized AuNPs and functionalized Type A, B, C, D Gd-DNA-AuNPs measured by DLS, with average hydrodynamic diameter of 7.102 nm, 13.54 nm, 17.03 nm, 11.64 nm and 19.32 nm for each type of AuNPs. The polydispersity index are calculated to be 0.2142, 0.06537, 0.1397, 0.1968, and 0.1474 for citrate AuNPs and Type A, B, C, D Gd-DNA-AuNPs respectively. **D**. Stability measurement of functionalized Type A, B, C, D Gd-DNA-AuNPs. 1/T1 (relaxation rate) is measured over a period of 2 years.

The successful functionalization of AuNPs across all four types were characterized by UV–vis spectroscopy (Fig. 2A). All functionalized GdAuNPs maintained a consistent surface plasmon resonance peak at 520 nm, indicating a preserved colloidal stability and core size following surface modifications. A decrease in Gd loading occurs in Type B due to the attachment of ssDNA. Type C exhibited the lowest Gd payload, as attachment of chelates to ssDNA is limited. A marked increase in gadolinium loading from Type C to Type D, as quantified by ICP-MS, was evident (Fig. 2B). Type D—incorporating both DNA-conjugated and directly attached Gd chelates—achieved a Gd loading as high as Type A, but with the additional functionality of ssDNA. These measurements validated our design rationale.

Hydrodynamic diameters measured by DLS showed systematic increases with each surface modification (Fig. 2C). Type A particles exhibited a hydrodynamic size of 13.54 ± 0.48 nm, increasing to 17.03 ± 0.78 nm for Type B due to ssDNA attachment. Type C had a slightly smaller hydrodynamic diameter (11.64 ± 0.52 nm), potentially due to reduced flexibility or folding of the Gd-chelate-labeled DNA, while Type D exhibited the largest diameter at 19.32 ± 0.94 nm, reflecting the highest surface loading (Supplementary Figure 6). DLS measurements indicated that sample concentrations did not affect GdAuNPs sizing (Supplementary Figure 7). The polydispersity index (PDI) was calculated to be 0.15 for citrate (i.e. blank) AuNPs, with similarly monodisperse distributions for Type A, B, C, D Gd-DNA-AuNPs (Table 3). DLS and PDI measurements indicate that no aggregation of GdAuNP occurred at higher concentrations. The zeta potential was measured to be −36.6 mV for citrate AuNPs and −41.63 mV, −29.99 mV, −43.11 mV, −44.23 mV for Type A, B, C, D Gd-DNA-AuNPs respectively. Long-term physicochemical stability is a critical parameter for biomedical nanoparticles. GdAuNPs of all types retained consistent 1/T_1_ relaxivity values over a two-year period (Fig. 2D), indicating excellent physicochemical stability. These results suggest that GdAuNP afford a high level of reproducibility and are suitable for use after long-term storage.

**Fig. 3.**
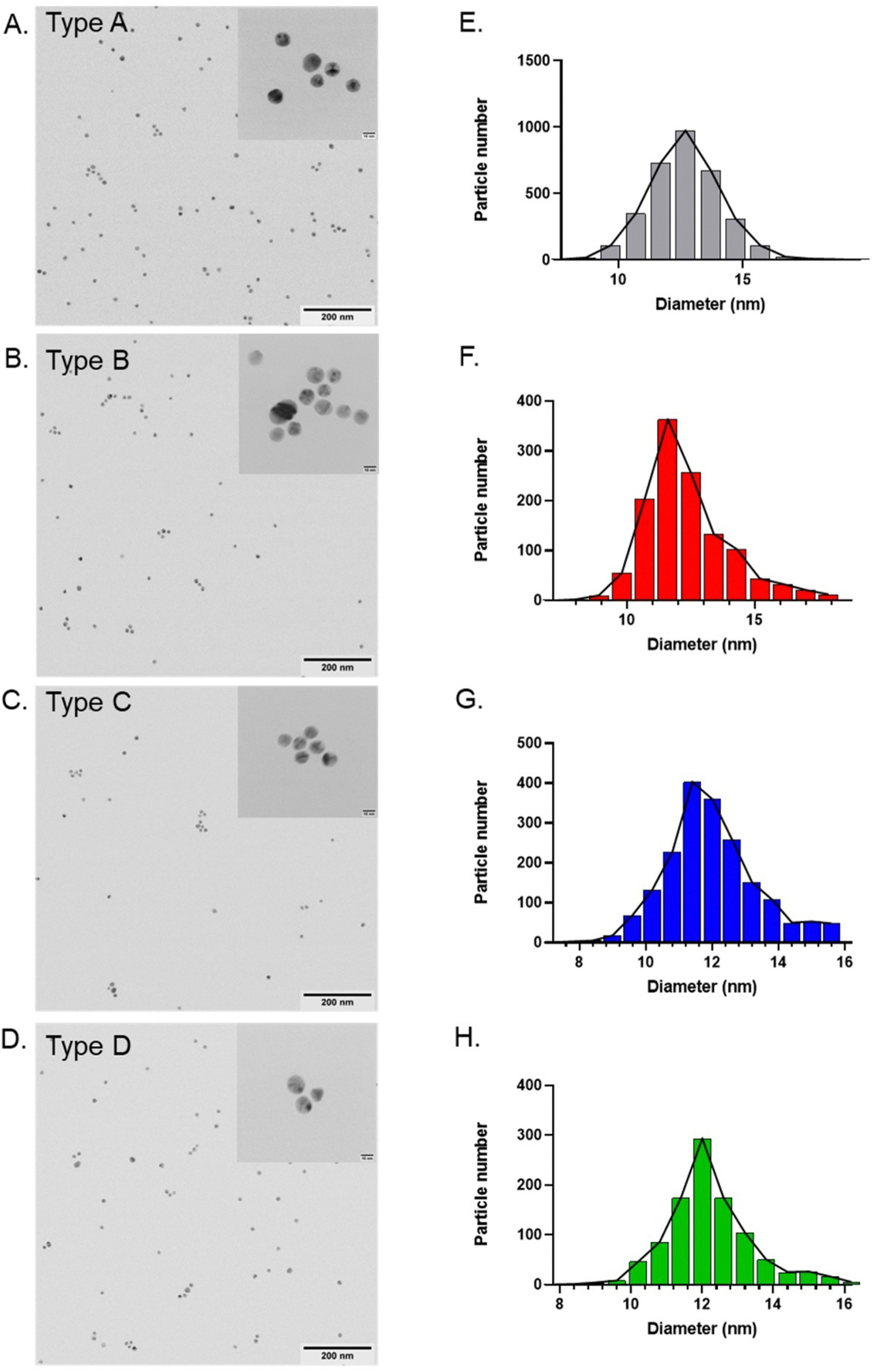
TEM and the corresponding histograms. **A-D.** Transmission electron microscopy (TEM) images of Type A, Type B, Type C, and Type D GdAuNP with an optimal concentration of 10 nM AuNPs at 100,000 × (scale bar = 200 nm) and 900,000 × (scale bar = 10 nm) magnification. **E-H.** The corresponding histograms of nanoparticles sizing based on TEM images for **E.** Type A Gd-AuNPs with average diameters of 12.2 nm and standard deviation of 1.48 nm (n = 3344). **F.** Type B Gd-DNA-AuNPs with average diameters of 12.0 nm and standard deviation of 1.71 nm (n = 1245). **G.** Type C Gd-DNA-AuNPs with average diameters of 11.7 nm and standard deviation of 1.44 nm (n = 1912). **H.** Type D Gd-DNA-AuNPs.

**Fig. 4.**
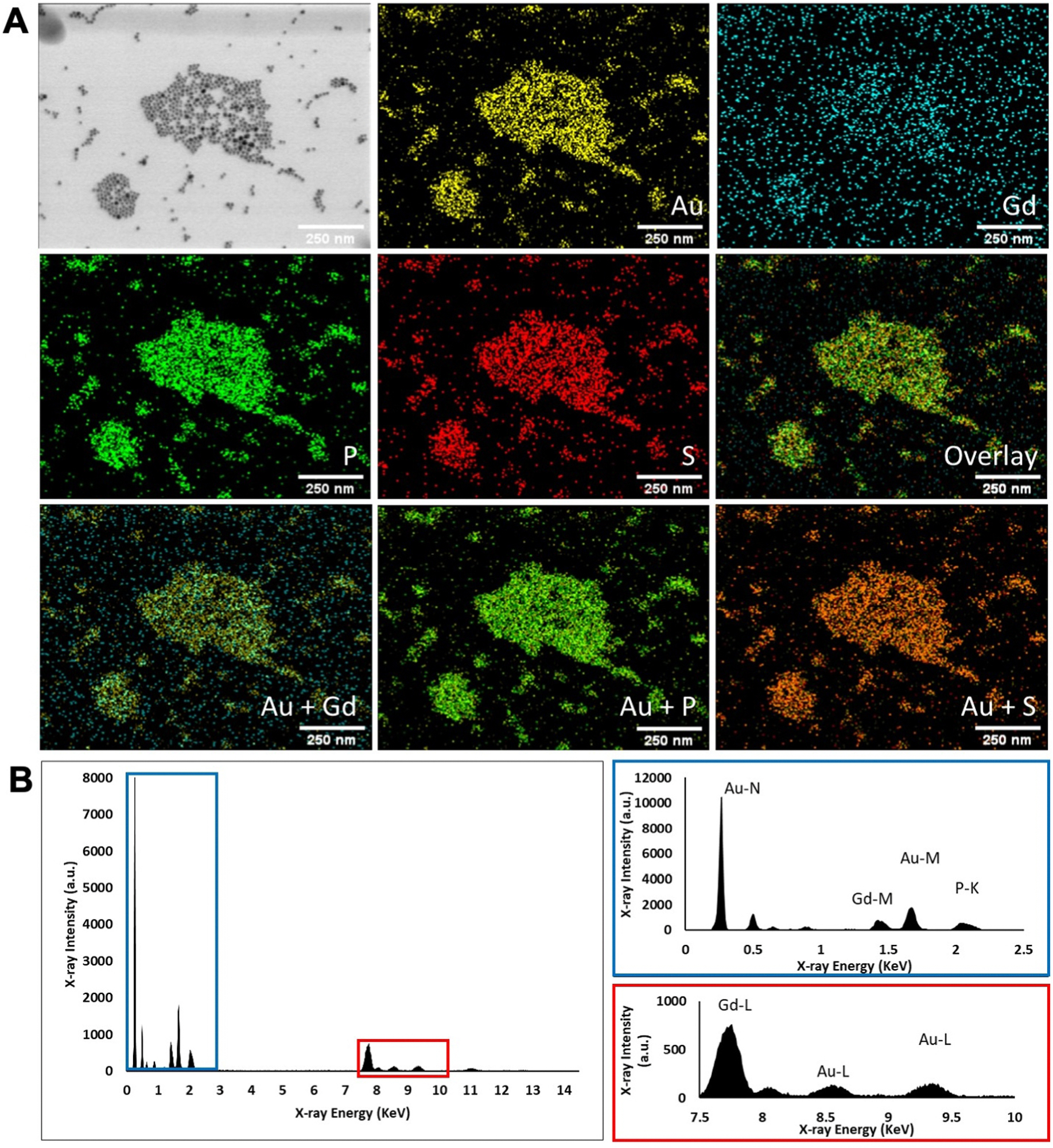
EDX elemental mapping & spectrum of GdAuNP composition. **A**. Compositional elemental mapping of GdAuNP, including bright field TEM image, Au, Gd, P and S layout that detected to have high percentage in sample, also shown in spectrum. Overlay of the elements and Au image is co-localized with Gd, P and S, represents the successful conjugation of ligand onto citrate stabilized AuNPs. **B.** Full X-ray spectrum for all elements detected. Extended spectrum for elemental analysis and peaks is assigned for Au, Gd and P.

TEM images taken with 10 nM GdAuNPs showed well-dispersed spherical nanoparticles across all formulations, with no observable aggregation or deformation (Fig. 3A–D). Higher concentrations (100 nM) GdAuNPs also did not reveal an aggregation or deformation (Supplementary Figure 8). Histogram analysis revealed tightly distributed core diameters centered around 12 nm: 12.2 ± 1.48 nm (Type A), 12.0 ± 1.71 nm (Type B), 11.7 ± 1.44 nm (Type C), and 11.9 ± 1.17 nm (Type D) (Fig. 3E–H). These values were consistent with expectations for 12 nm citrate-AuNPs, and the slightly narrower distribution in Type D may reflect steric stabilization from dense ligand packing. The marginal size increase is attributed primarily to the flexible ssDNA and chelate layers, which add hydrodynamic volume without altering the AuNP core. Elemental mapping via energy-dispersive X-ray spectroscopy (EDX) further validated the co-localization of Au with Gd, phosphorus (from DNA), and sulfur (from thiolated linkers), confirming successful and multi-layered conjugation (Fig. 4A). Overlay images demonstrate uniform elemental distribution, while full X-ray spectra (Fig. 4B) clearly identified peaks corresponding to each expected element. This supports the compositional integrity of our multivalent design strategy.

Taken together, this systematic, stepwise assembly of GdAuNPs demonstrated a predictable and tunable control over particle size, gadolinium loading, and chemical structure. The ability to co-functionalize particles with optical tags and ssDNA opens avenues for multimodal tracking and programmable targeting in biological systems. These physicochemical characteristics form the foundation for *in vivo* studies addressing the *in situ* cellular uptake of GdAuNP and MRI/CT contrast.

### 3.2. MRI relaxometry of GdAuNP types

To ensure that contrast agents were not affected by transportation between the production and application sites, relaxivity was measured before and after shipping. In all cases, relaxivity was not impacted by shipping (Supplementary Fig. 9). To provide a detailed characterization of the MR relaxivity and μCT signal attenuation properties of different GdAuNP, a series of concentrations were prepared in NMR tubes and compared to vehicle controls (Supplementary Fig. 10). Qualitatively, T1w contrast required different concentrations to afford a visual detection of a hyperintense signal (ProHance 66 μM, Type A 100 μM, Type B 250 μM, Type C 500 μM, Type D 50 μM). Quantitatively, this T1w signal change accounted for a 30 % increase compared to the vehicle control. Any T1w contrast increase that was <30 % was not visually discernible.

**Fig. 5.**
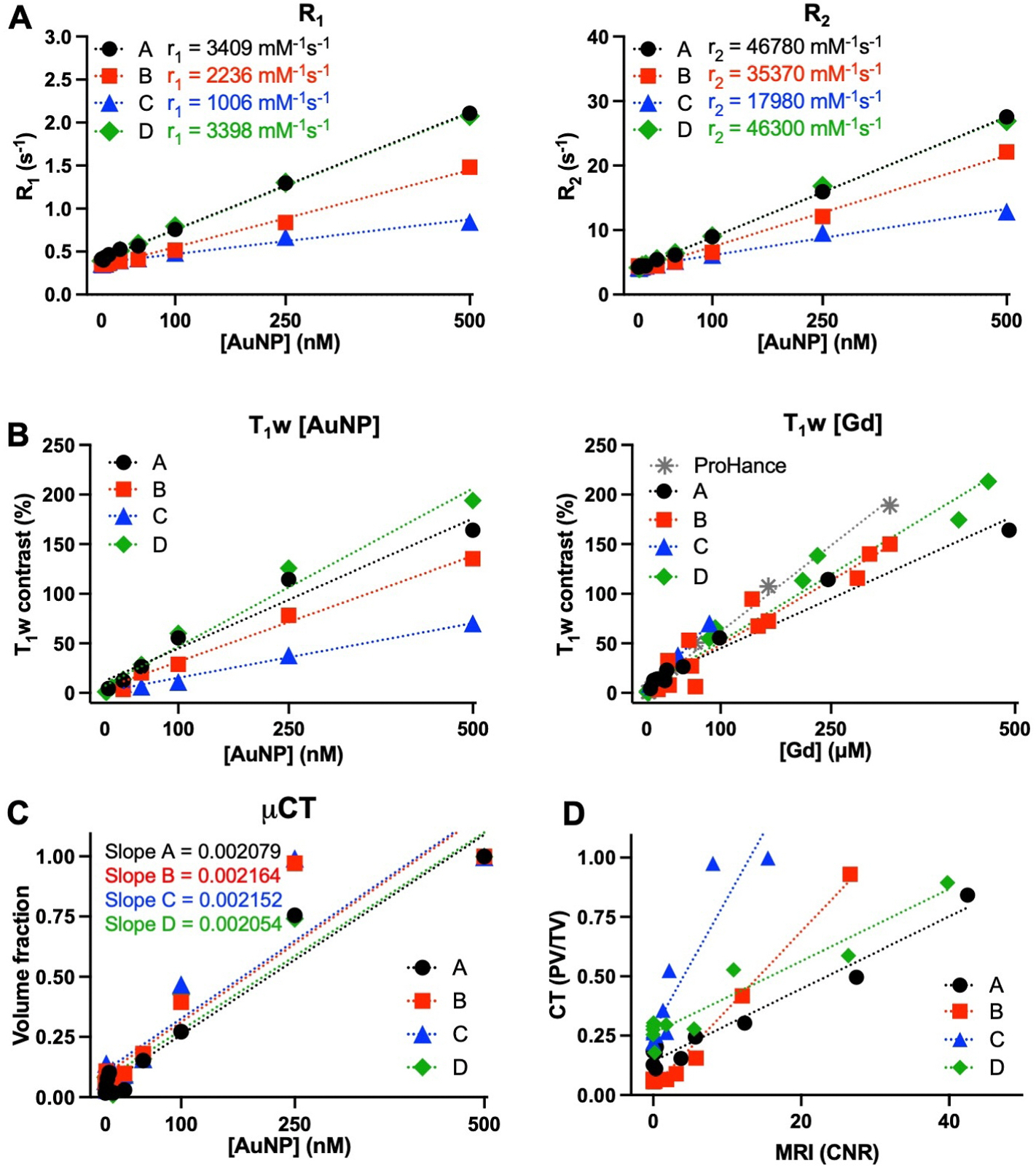
MR Relaxometry and μCT signal attenuation of GdAuNP in solution. **A**. The T1 and T2 MR relaxometry for Type A-D GdAuNP for solution concentrations up to 500 nM (n = 3/condition). Types A and D exhibited the greatest T1 relaxivities. However, Type D GdAuNP were the most efficient in producing T_1_-weighted (T_1_w) contrast (i.e. hyperintense signal) per AuNP (**B**), likely due to the slightly more favorable r_2_. When considering Gd loading, Type D also exerted a high per Gd relaxivity, but this was slightly reduced compared to a standard monomeric Gd particle (i.e. GdHPDO3A, ProHance). **C**. μCT of the same GdAuNP solutions also exhibited a linear relationship between AuNP concentration and volume fraction on μCT images. **D**. Correlational analysis between contrast-to-noise on MR and μCT images indicated a high level of consistency in signal change in both imaging modalities using GdAuNP. Type C achieved a higher signal attenuation in MRI than μCT as indicated by a steeper linear regression curve.

R1 (1/T1) and R2 (1/T2) were highest for Type A and Type D (Fig. 5A), both having Gd directly conjugated to the AuNP as a design characteristic (Table 4). The longitudinal relaxivity (r1) for Type A GdAuNP (based on AuNP concentration) was 3409 mM^−1^s^−1^ (Table 5). Type B, followed by Type C, had lower r_1_ values. Type C exhibited the lowest r_1_ at 1006 mM^−1^s^−1^ (per AuNP). The lower T_1_ relaxivity of Type C is due to the much lower Gd loading of these particles compared to Type A and D (Table 5). Interestingly, Type C had the highest r_1_ for Gd at 5.934 mM^−1^s^−1^, which was higher than the standard ProHance relaxivity (5.165 mM^−1^s^−1^). All other GdAuNP designs had lower r_1_ for their Gd concentration. T_2_ relaxivity (r_2_) followed a similar pattern than observed for r_1_. The r_2_ for Gd was enhanced more than 10x for Type A, B and D and almost 20x for Type C.

As expected from the relaxivity measurements, T1w contrast was highest with Type D and Type A designs based on AuNP concentrations (Fig. 5B). However, Type C had the highest T1w contrast when considering Gd loading. By applying the 30 % T1w contrast detection threshold, the AuNP content required to achieve a visual detection of a signal hyperintensity is 52 nM for Type D (highest r1) and 218 nM for Type C (lowest r1). In contrast, a Gd loading of 33 μM is required for Type C (highest r1) and 59 μM for Type A (lowest r1).

The Gd loading on GdAuNP varies depending on the design characteristics. However, the AuNP size and density is consistent. For μCT imaging, it is expected that the AuNP size/content is the determining factor of signal attenuation. Indeed, there was a linear relationship between AuNP concentration and volume fraction on μCT images, with all GdAuNP designs having very similar signal attenuation characteristics (Fig. 5C). This demonstrates that Gd content only had a minor, possibly negligible, contribution to μCT detection. A correlational analysis between MRI CNR and signal attenuation on μCT revealed a strong relationship between both imaging modalities (Type A r = 0.98, p < 0.001; Type B r = 0.99, p < 0.001; Type C r = 0.92, p < 0.001; Type D r = 0.96, p < 0.001, Fig. 5D). These results further highlight that Au primarily defined μCT detection, whereas Gd content primarily determined MR relaxivity.

### 3.3. Stereotactic injections of GdAuNP

The intracerebral injection of GdAuNP is achieved through a 32 gauge (G) metal needle. To ensure that GdAuNP do not react with the ferrous needle, potentially accumulating within the barrel or being retained within the needle, a series of experiments were conducted to measure how much Gd and Au was retained within the needle after ejection. A significant amount of Gd and Au was retained within the needle itself after ejection of GdAuNP was completed (Supplementary Fig. 11). The highest amount of Gd and AuNP was, however, retained on the surface of the needle, with the cleaning solution (Hamilton) removing most of this. Washes indicated that the amount of retained GdAuNP was reduced 20x by the cleaning solution. A negligible amount of iron was washed out of the needles. However, in the context of MRI this could lead to minute depositions of iron within tissues. It is also important to note that there was no difference in Gd and Au content based on how many passes of ejection were performed, indicating that no gradual increase of Gd or Au in the needle occurred.

To verify the stereotactic accuracy of injections and ensure that GdAuNP provide a robust T1w contrast *in situ*, rats were injected post-mortem (straight after perfusion fixation) with GdAuNP into the right lateral ventricle (Supplementary Figure 12A). All designs of GdAuNP produced a T_1_w signal enhancement, with the strongest signal changes evident within the injected ventricle with Type B and D. As there is no CSF flow in these animals, GdAuNP are not biologically cleared and afford robust MR imaging over time. For *ex vivo* post-mortem imaging, a key question is whether these GdAuNP remain in a stable location or if these distribute through the ventricular system and possibly permeate into the surrounding parenchyma. Serial MR imaging of rat heads indicated that over 30 days post-injection a slow, but a gradual shift in GdAuNP occurred through the ventricular system with some permeation into neighboring tissue (Supplementary Figure 12B). Post-mortem MR imaging of GdAuNP injected into the lateral ventricles is hence best performed within a couple of weeks post-injection.

### 3.4. Impact of tissue environment on GdAuNP detection

**Fig. 6.**
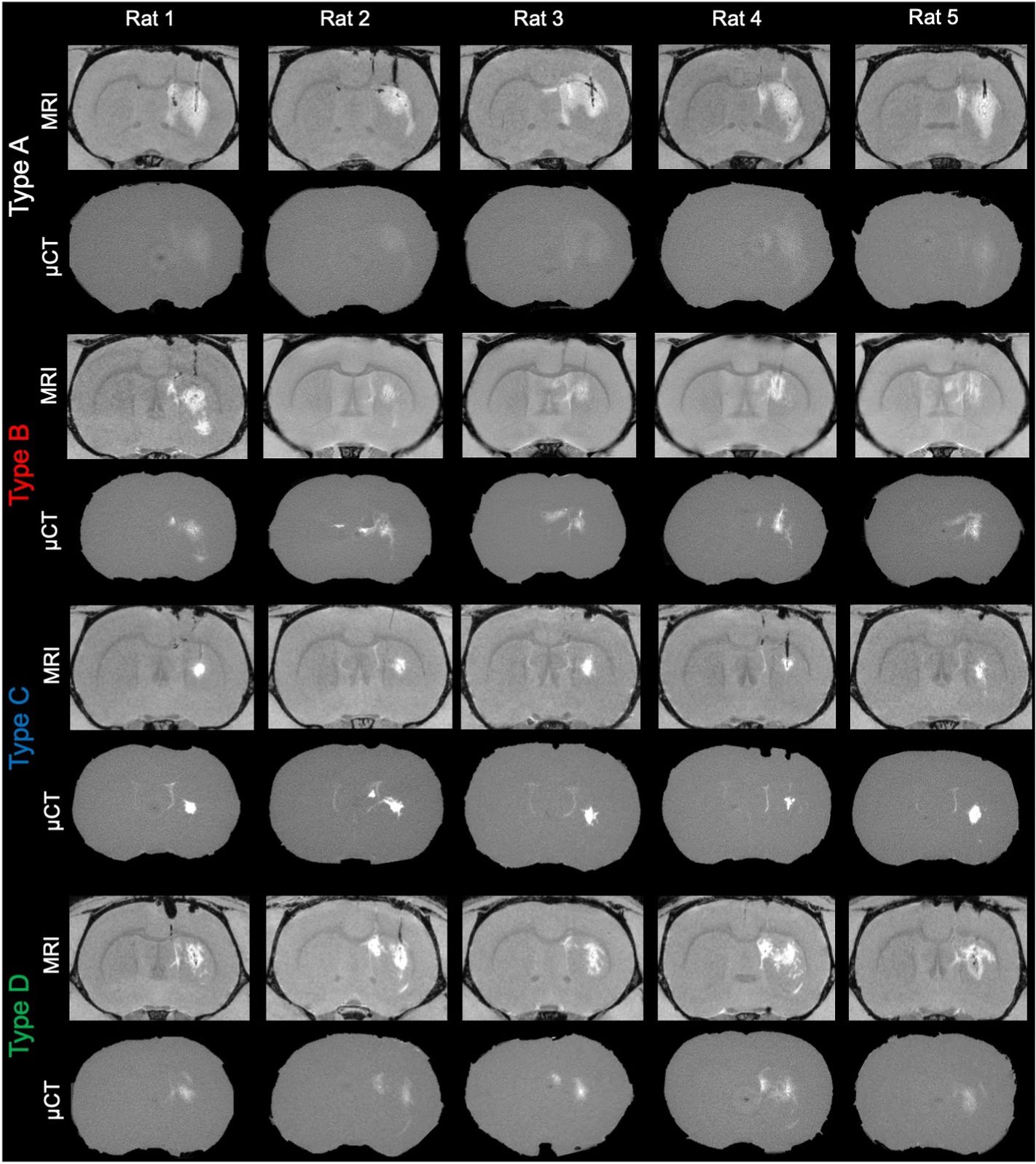
Impact of tissue environments on MRI and CT contrast. Injection of GdAuNPs (500 nM) into the lateral ventricle and the striatum revealed high T1w MRI contrast in all animals. However, qualitatively some GdAuNP (Type A) distributed over larger areas of the striatum than others (Type C). Although there was a high consistency in MRI and μCT signal detection, the MRI hyperintensities were more robustly detected than the μCT signal attenuation.

To determine the impact of an aqueous versus tissue environment for GdAuNP detection, the 4 different GdAuNP design types were injected into rats’ right lateral ventricle, as well as striatal tissue. After 24 h of survival, *ex vivo* MRI afforded the detection of GdAuNP in all conditions (Fig. 6). μCT also detected GdAuNPs in all animals, but signal attenuation was less prominent than in MRI. It is noteworthy that GdAuNP Type C exerted the highest contrast on both MRI and μCT, but that the distribution of GdAuNP was the lowest. In contrast, Type A GdAuNP distributed the widest and achieved a low signal attenuation on μCT, potentially reflecting a decreased voxel concentration.

**Fig. 7.**
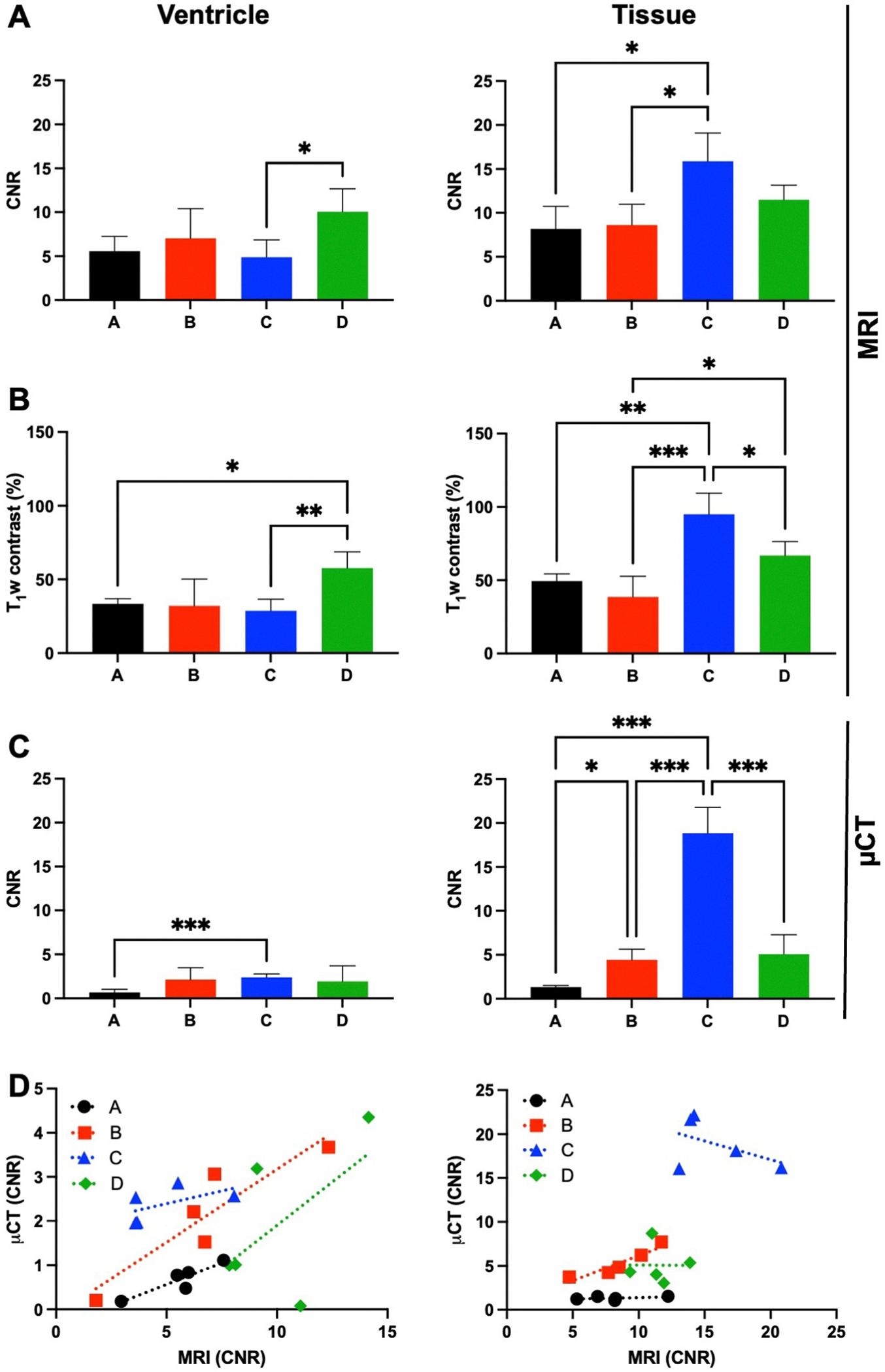
Quantification of tissue contrast. **A**. To compare the contrast-enhancing properties of GdAuNP types (n = 5/condition) in an aqueous and parenchymal environment, Contrast-to-noise and T1w contrast was measured in the ventricle and striatum, respectively. Within the ventricle, Type D GdAuNP exerted the most significant increase in CNR and T1w contrast, whereas within the striatum Type C achieved the highest increase. Both Type A and B achieved equivalent contrast, but significantly lower than Type C and D. **B**. CNR on μCT was most significantly attenuated for Type C in both the ventricle and striatal tissue. However, a major difference in signal attenuation was evident between both microenvironments, with parenchymal contrast being much higher than ventricular signal attenuation. **C**. A Pearson correlation between MRI and μCT revealed generally a positive linear relationship between both modalities for both ventricular and parenchymal environments. However, Type C exhibited a negative correlation in the striatum, mostly due to the high signal attenuation of these GdAuNP on μCT. (*p < 0.05; **p < 0.01, ***p < 0.001).

Quantification of signal intensity of GdAuNP in relation to noise (i.e. contrast-to-noise, CNR) indicated that both Type A and B achieved similar increases in signal intensity. This was evident in both the ventricles, as well as striatal tissue (Fig. 7A). Type C achieved a similar CNR in the ventricles, with a significantly increased CNR in tissue (p < 0.05). Type D performed equally well in both ventricles and tissue, having the highest CNR in the ventricles compared to all design configurations (p < 0.05), but being marginally inferior to Type C in striatal tissue. T1w contrasts followed the same pattern of results than CNR, but provided more robust statistical differences between conditions. Type D GdAuNP overall achieved the most efficient signal increase in both ventricle and tissue.

Signal attenuation on μCT indicated that Type A GdAuNP were the least efficient design (p < 0.001, Fig. 7B), whereas Type C achieved the highest signal attenuation in both ventricle and striatum (p < 0.001). The signal attenuation in the striatum was markedly (x3.5) higher compared to other GdAuNP designs. This is likely a reflection of the more concentrated distribution of the particles compared to the more distributed nature of, for instance, Type A GdAuNP. There was a good correlation between the degree of MRI signal intensity changes and signal attenuation on μCT in the ventricles and striatal tissue (Fig. 7D). Within the ventricles, Type B exhibited the highest correlation (r = 0.913, p < 0.05), followed by A (r = 0.909, p < 0.05), D (r = 0.576, p = 0.15), and C (r = 0.564, p = 0.16). Within striatal tissue, however, a more complicated relationship emerged. Type B exhibited a very strong correlation between MRI and μCT (r = 0.939, p < 0.01), whereas Type A (r = 0.423, p = 0.24) and D (r = −0.003, p = 0.5) did not show any correlation. Type C exhibited a non-significant negative correlation (r = −0.469, p = 0.21).

**Fig. 8.**
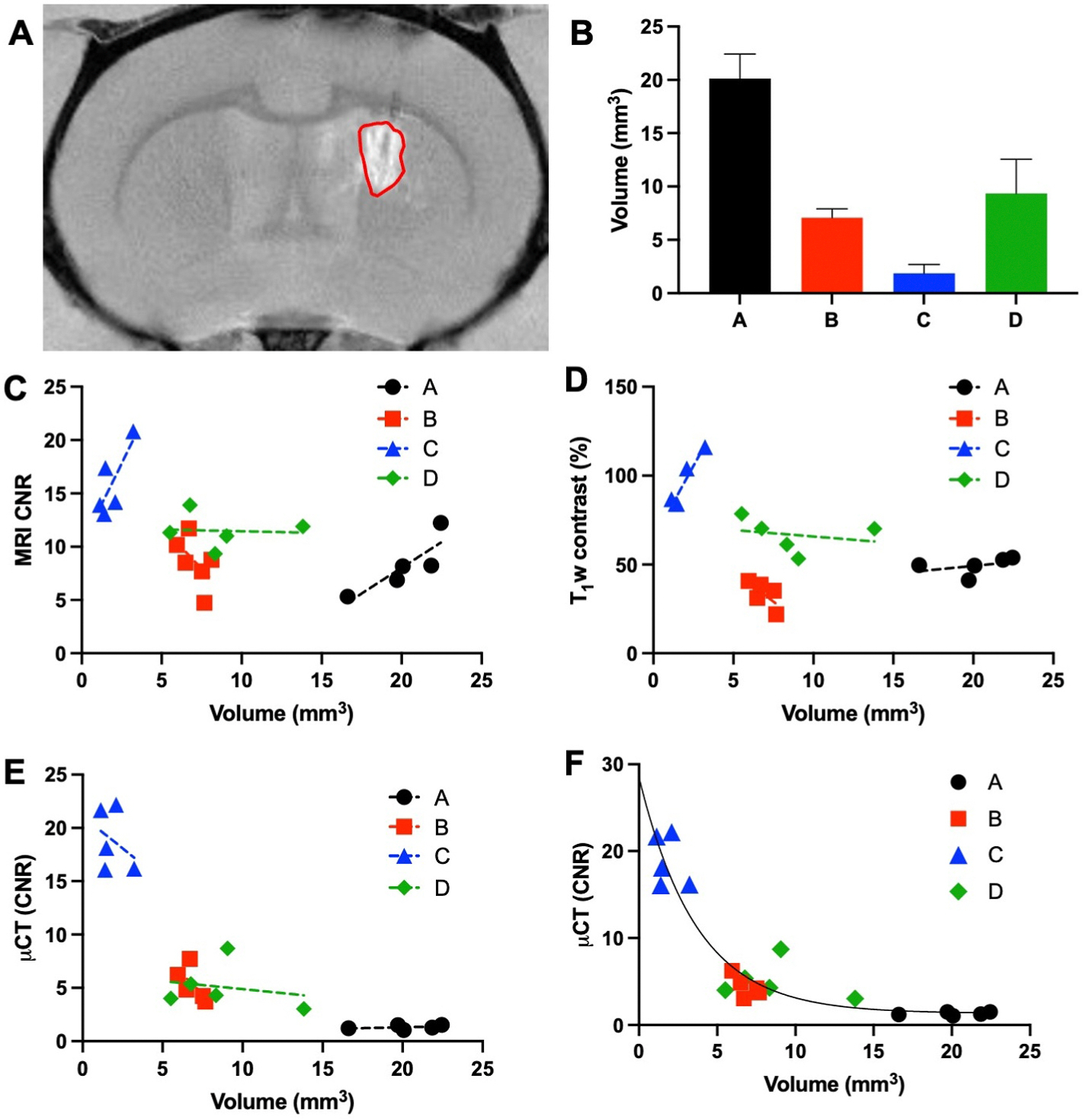
GdAuNP distribution and intrastriatal contrast. **A.** Illustration of region-of-interest (ROI) drawn to measure T1w signal distribution in the striatum. **B**. Type A GdAuNP were most widely distributed in the striatum, whereas Type C GdAuNP were least distributed. **C**. Correlational analysis of GdAuNP volume distribution with change in CNR on MR images reveal that a tighter distribution, as observed with Type C, achieves a higher CNR. Volume and contrast for Type C was highly correlated, but other GdAuNP, such as Type D, did not exhibit an individual correlation between volume and CNR. **D**. Akin to CNR, T_1_-weighted (T_1_w) contrast change was associated with volumetric distribution. However, Type A achieved a slightly higher T1w contrast over a larger distribution area than Type B, indicating that volumetric distribution by itself is not sufficient to determine contrast efficiency. **E**. μCT revealed similar efficiencies of GdAuNP in relation to their volumetric distribution with Type C achieving the strongest contrast, as particles were distributed over a much smaller volume compared to all other designs. Type A which was most widely distributed achieved the lowest contrast on μCT after intrastriatal injection. **F**. Fitting of GdAuNP contrast with their volume of distribution on μCT images can be described using a polynomial regression curve.

The volume of tissue distribution can dilute the local signal intensity induced by contrast agents. Measurement of distribution volume can hence provide important insights into signal intensity measurements (Fig. 8A). Type A covered a volume ~4x as large as Type B and D, but >10x larger than the distribution of Type C (Fig. 8B). The more distributed nature of Type A hence reduces its signal intensity at the voxel level, whereas the more confined distribution of Type C increases its local signal intensity, as reflected in correlations between MRI-based CNR (Fig. 8C) or T_1_-weighted signal intensities (Fig. 8D) with volume of distribution. CT followed the same relationship between volume of contrast agent distribution and CNR (Fig. 8E) and revealed a loss function curve that clearly indicated that a higher signal intensity is related to a narrower distribution (i.e. higher local contrast agent concentration, Fig. 8F). The environment and distribution within which GdAuNP reside hence influences their detection using different imaging modalities.

### 3.5. In situ cellular uptake of GdAuNP types

**Fig. 9.**
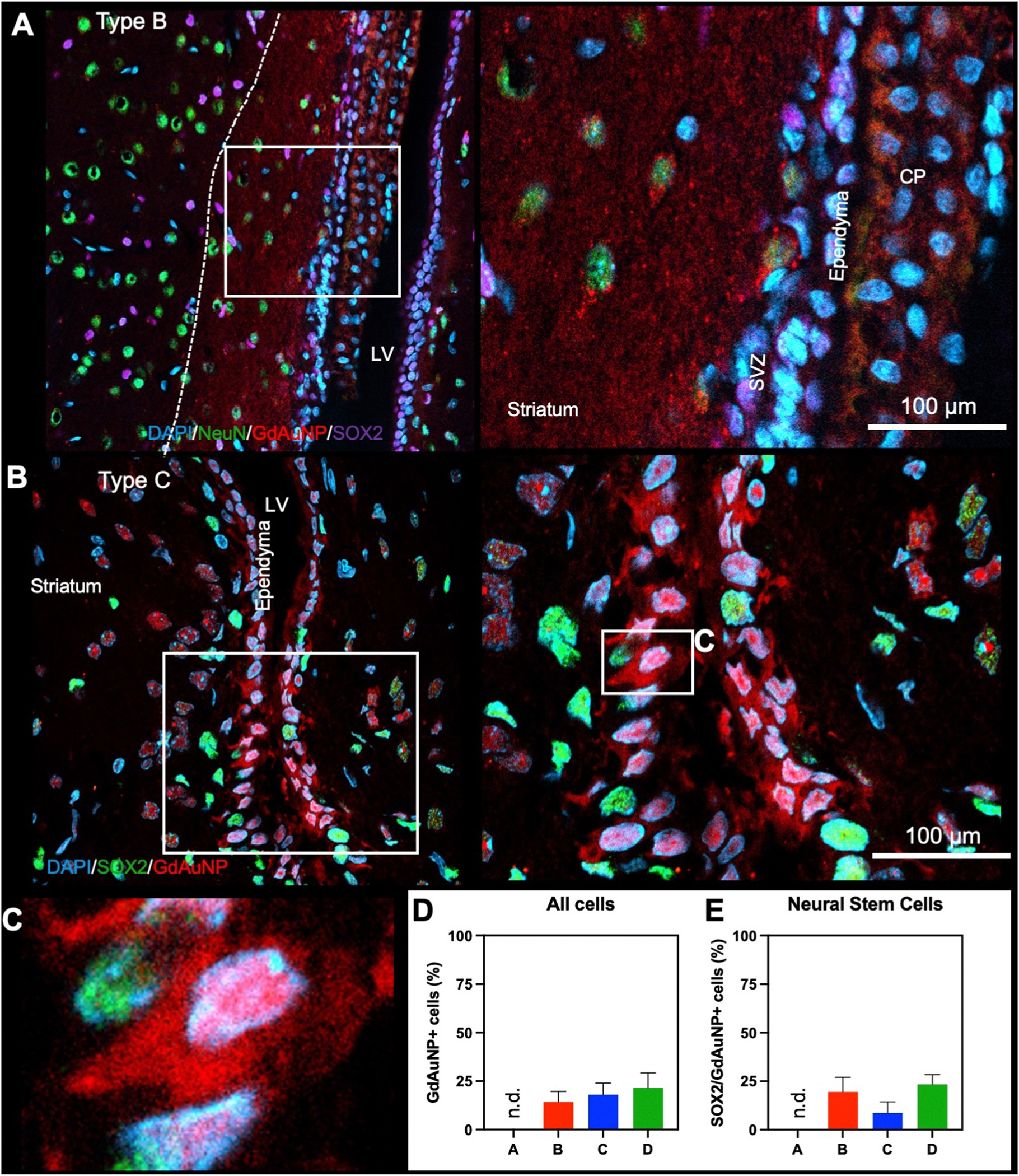
Intracerebroventricular injections of GdAuNP and cellular uptake in the subventricular zone (SVZ). **A**. Immunohistochemical analysis of GdAuNP injected into the lateral ventricles (LV) revealed a permeation into the SVZ and into the striatum. Cells within the choroid plexus (CP, located inside the LV) took up GdAuNP (in red). SOX2+ neural progenitor cells (NPCs) within the SVZ took up GdAuNP, as did NeuN+ neurons in the neighboring striatum. At this middle level of the LV, the ependymal cells lining the ventricular wall were only weakly labeled with GdAuNP. **B**. In contrast, in more anterior regions of the LV, ependyma were highly labeled with GdAuNP. **C**. GdAuNP in ependymal cells (24 h post-injection) were present within the cytoplasm, as well as in the peri-nuclear space. **D**. Quantification of GdAuNP retained within tissue sections (n = 5/condition), indicated a consistent cellular labeling for all ssDNA-GdAuNP (~20 % of all cells). **E**. Within the SVZ, Type B and D achieved a similar labeling of SOX2+ NPCs, whereas Type C was less efficient in labeling these cells. (For interpretation of the references to color in this figure legend, the reader is referred to the Web version of this article.)

Within the tissue environment, distribution/permeation through the parenchyma, cell uptake, as well as interactions with proteins can affect the relaxivity of GdAuNP. A key feature of GdAuNP is their cell membrane penetration and uptake into non-phagocytic cells. To evaluate cell uptake *in situ*, GdAuNP were injected intracerebroventricularly leading to their uptake into cells of the SVZ (Supplementary Figure 13). The SVZ is lined with a single layer of ependymal cells, as well as the neurogenic niche, which is characterized by NPCs (SOX2+). Type B, C, and D exhibited uptake into cells, mostly NPCs (Fig. 9A), but some uptake into ependymal cells was also evident in more anterior regions (Fig. 9B). GdAuNP were widely distributed throughout the cell's cytoplasm at 24 h but tended to accumulate around the nucleus (Fig. 9C). To ensure that the fluorescence signal is indeed from the GdAuNP, SEM imaging was used to verify the distribution of nanoparticles in brain tissue slices, further confirming that indeed AuNP were accumulating within cells of the SVZ (Supplementary Figure 14). Although Type A permeated through the tissue, it typically remained outside of cells and was washed off during immunohistochemistry processing (Supplementary Figure 15). It hence could not be included in quantifications.

In the main chamber of the lateral ventricles, ependymal cells were not typically taking up significant amounts of GdAuNP, but GdAuNP were distributed along the peri-ventricular region in a gradient tapering off into striatal tissue. Wherever GdAuNP permeated into tissue, it was taken-up by cells. Within the SVZ, between 15 and 20 % of cells were labeled with GdAuNP, but no significant difference between nanoparticle design was evident (Fig. 9D). Type B and D labeled approximately twice as many SOX2+ cells (~20 %) than Type C (Fig. 9E). These results hence indicate that GdAuNP are robustly endocytosed by NPCs in the SVZ, as well as in some circumstances ependymal cells of the anterior horn of the lateral ventricles.

**Fig. 10.**
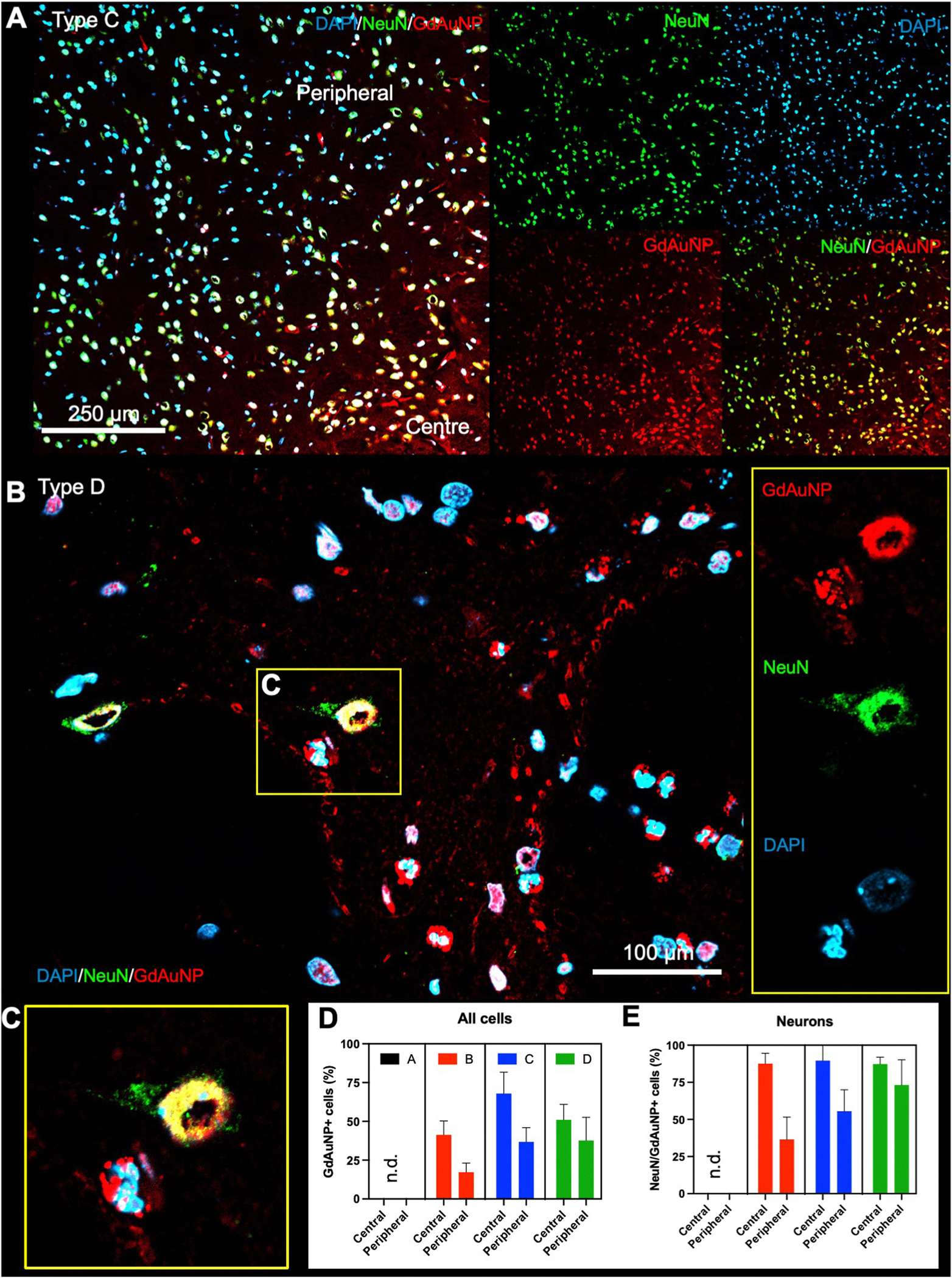
Intra-striatal injections of GdAuNP and cellular uptake into striatal neurons. **A**. Immunohistochemical analysis of the *in situ* labeling of striatal cells with GdAuNP. At the centre of striatal injections, a higher concentration of GdAuNP were present, whereas in peripheral areas, a lower concentration was evident, as revealed by the red fluorescence intensity of GdAuNP. An extensive uptake of GdAuNP was evident in NeuN+ neurons, especially at the centre of injection. **B**. GdAuNP were endocytosed by neurons, as well as other cells, and typically accumulated around the cell's nucleus. **C**. GdAuNP created a halo around the nucleus of striatal cells. **D**. Quantification of cellular uptake indicated a higher endocytosis in the central region of injections, whereas peripheral areas with a lower GdAuNP exhibited less uptake (n = 5/condition). Type C was the most efficient design for cellular uptake in the striatum. **E**. Within the central region, GdAuNP were very efficient to label neurons, with >80 % containing GdAuNP. Within peripheral regions, the Type D design was most efficient to warrant uptake into neurons, whereas Type B was the least efficient. (For interpretation of the references to color in this figure legend, the reader is referred to the Web version of this article.)

Within striatal tissue, the contrast agent distribution and hence local concentration affect cell uptake (Supplementary Figure 16). SEM imaging further verified the presence and distribution of discrete, electron-dense particles within the striatum, whereas *in situ* mapping by EDX demonstrated the co-localization of the gold (Au) signal within these particles, confirming their identity as Au-containing GdAuNPs (Supplementary Figure 17). At the injection site, a high concentration of contrast agent is available, but as this permeates through the tissue, lower concentrations are present in peripheral regions (Fig. 10A). Uptake of GdAuNP was preferentially into neurons (Fig. 10B), with a peri-nuclear localization (Fig. 10C). A higher proportion of cells contained GdAuNP at sites central to the injection, whereas significantly fewer cells were labeled at peripheral sites (Fig. 10D). Type C was the most efficient design to label non-dividing cells (~68 %), followed by Type D and Type B. Local availability of contrast agent hence defined labeling efficiency. Over 80 % of neurons contained GdAuNP of all types at central sites (Fig. 10E). At peripheral sites, Type D was most efficient, whereas Type B was least efficient to label NeuN + cells (i.e. mature neurons), suggesting that particle design is an important factor in neuronal uptake at lower contrast agent concentrations.

**Fig. 11.**
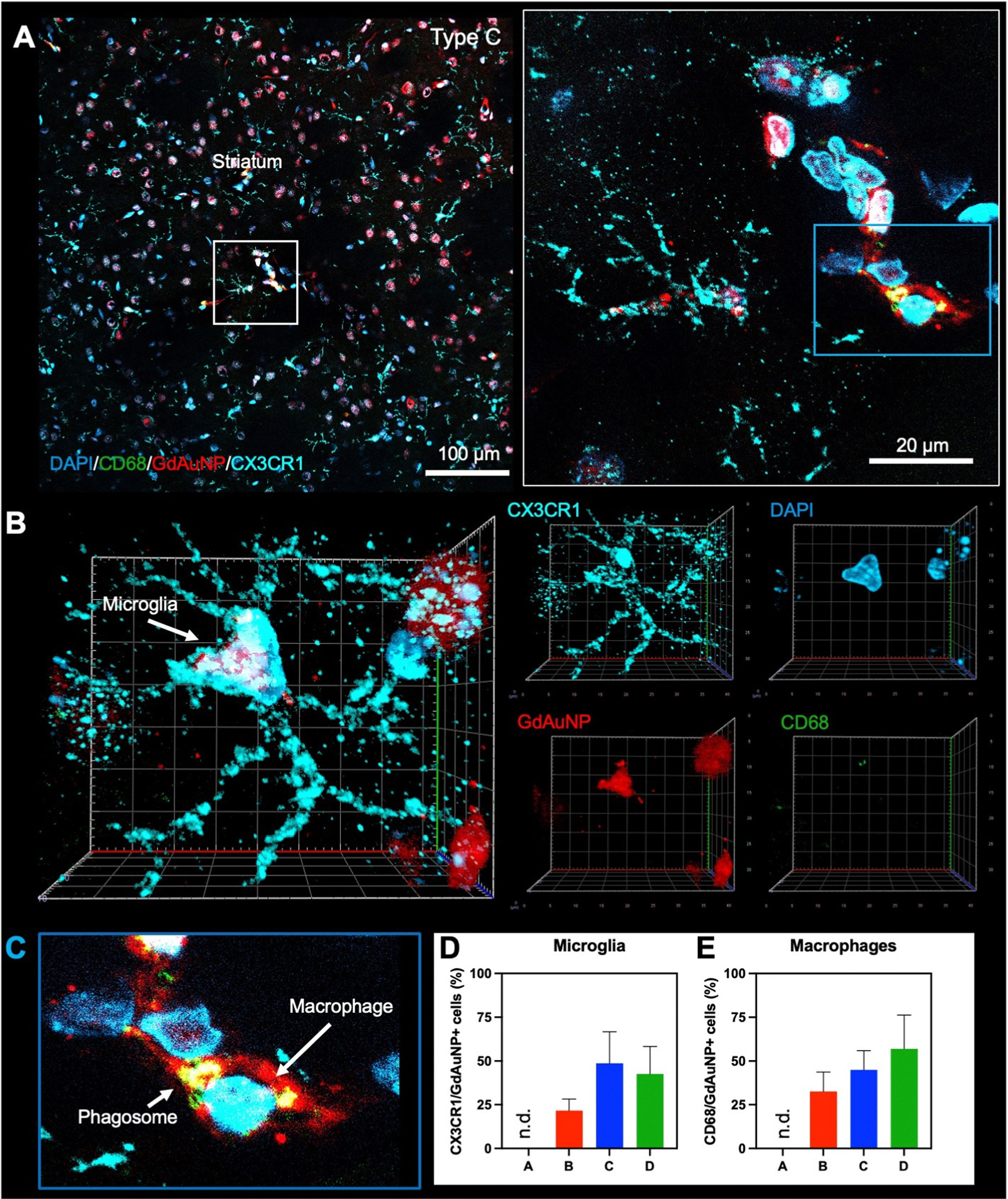
GdAuNP uptake into microglia/macrophages. **A**. Immunohistochemical analysis of GdAuNP in microglial and macrophages. Only a few macrophages were present within the injection tract, whereas microglia were widely distributed throughout the tissue. GdAuNP were present within both types of immune cells. **B**. Within microglia, GdAuNP accumulated around the nucleus, but they were also present to a lesser and in a more sporadic fashion in processes. Uptake of GdAuNP from the extracellular space through cellular processes is assumed to lead to intracellular transport to a peri-nuclear accumulation. **C**. In macrophages, the intracellular distribution of GdAuNP was distinct from other cells, as very little peri-nuclear accumulation was observed. GdAuNP were mostly contained within phagosomes indicating a phagocytic rather than endocytic cellular uptake. **D**. Type B was least incorporated into microglia (n = 5/condition), whereas Type C and D achieved a similar microglia uptake (~40 %). **E**. A similar pattern of uptake was evident in macrophages, with over 50 % of cells incorporating Type D GdAuNP, but only ~30 % of macrophages contained Type B GdAuNP.

In addition to neurons, GdAuNP were taken-up into phagocytic cells. Microglia (CX3CR1+) are resident phagocytic cells within the brain, whereas peripheral macrophages (CD68+) respond to tissue damage in the brain. Within the injection site, both types of immune cells are present, although microglia outnumbered macrophages. Only a few macrophages (<30 cells) were present at the site of injection damage. GdAuNP can be found in both types of cells (Fig. 11A). Within microglia (mostly of a resting phenotype), GdAuNP were present in the peri-nuclear space, but some GdAuNP were also located within processes (Fig. 11B), whereas within macrophages GdAuNP were located predominantly within phagosomes (Fig. 11C). Processes in activated microglia can take-up GdAuNP distributed throughout the tissue. Within macrophages, less localization to the peri-nuclear space was observed compared to other cells. Quantification indicated that Type C and D were most commonly taken-up by microglia (Fig. 11D), whereas Type D were more often present within macrophages (Fig. 11E). These results indicated that GdAuNP are mostly endocytosed by microglia, but are phagocytosed by macrophages. It is noteworthy that microglia and macrophages were less labeled with GdAuNP than neurons.

**Fig. 12.**
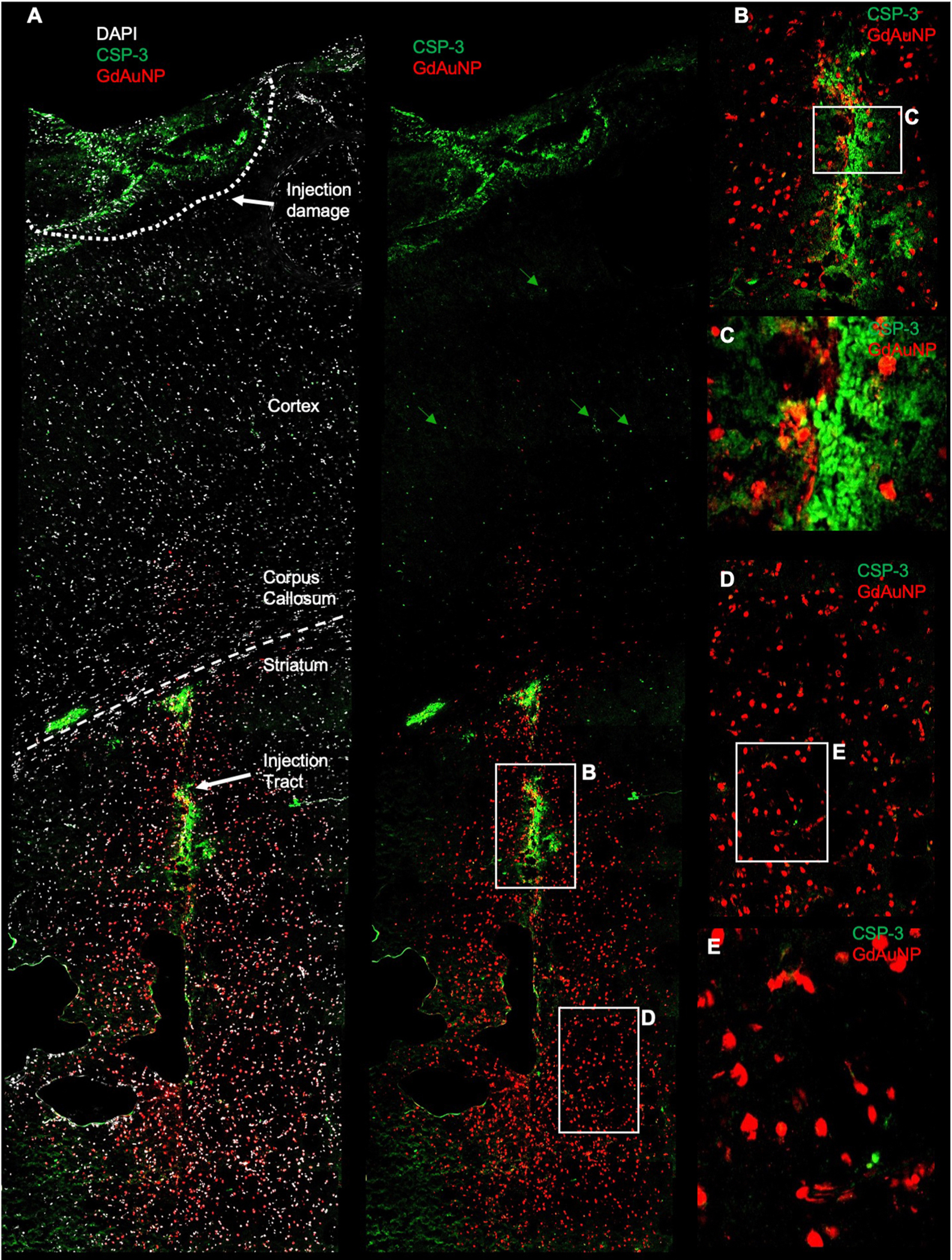
Caspase-3 expression. **A**. Caspase-3 (CSP-3), a common marker of apoptosis, is expressed in areas of injection damage, notably the surface of the cortex and the injection tract in the striatum. A low infrequent CSP-3 expression was also evident post-perfusion fixation in undamaged tissues, such as the cortex (green arrows). **B**. Cells around the inject tract contained GdAuNP (Type D), as well as in some areas CSP-3. **C**. High magnification confirmed that that some of the GdAuNP containing cells underwent apoptosis, but non-GdAuNP containing cells also underwent apoptosis. **D**. In areas outside the injection tract, large numbers of cells contained GdAuNP, but were not expressing CSP-3. **E**. Higher magnification further confirmed this observation, although occasionally CSP-3 expressing cells were observed. The incidence of CSP-3 cells in GdAuNP-labeled and unlabeled cells were very low (<1 %) in non-damaged tissues. (For interpretation of the references to color in this figure legend, the reader is referred to the Web version of this article.)

Uptake of GdAuNP into cells *in situ* did not induce caspase-3 (CSP-3) expression, indicative of programmed cell death (i.e. apoptosis). CSP-3 expression was consistently observed at the site of cortical penetration by the injection needle, but no GdAuNP were found at this site (Fig. 12A), indicating that this expression is purely related to iatrogenic injury. The undamaged cortex exhibited an infrequent (<1 % of cells) expression of CSP-3 in cells. The injection tract in the striatum also expressed high levels of CSP-3, narrowly confined around and along the tissue damage caused by needle insertion. GdAuNP were widely present in the cells in this area (Fig. 12B), including in apoptotic cells (Fig. 12C). However, outside areas of damage GdAuNP did not exhibit a CSP-3 expression (Fig. 12D) and were consistent with the low level of CSP-3 expression in this tissue post-perfusion fixation (Fig. 12E). GdAuNP of any design type did hence not induce an acute cytotoxicity *in situ*.

## 4. Discussion

*In situ* labeling of cells with contrast agents is essential for enabling cellular MRI [2,32]. In this study, we demonstrate that four distinct designs of gadolinium-functionalized gold nanoparticles (GdAuNPs) can be reproducibly synthesized, transported, and applied, while maintaining strong T_1_-weighted (T1w) contrast at a high magnetic field strength (9.4 T). The simplest formulation—Type A GdAuNPs—consisted of Gd chelates adsorbed onto the AuNP surface. These exhibited broad tissue dispersion, but minimal T1w contrast due to limited cellular uptake.

To improve uptake, polyT single-stranded DNA (ssDNA) was added (Type B), resulting in enhanced *in situ* internalization and stronger T1w contrast, though with reduced tissue distribution. In Type C, Gd chelates were covalently incorporated into the ssDNA, rather than directly attached to the AuNP surface. This design increased water accessibility to the Gd centers, thereby enhancing relaxivity. Type C GdAuNPs showed the highest localized T1w contrast and efficient neuronal labeling, albeit over a more limited spatial extent. Type D GdAuNPs combined the DNA-conjugated Gd of Type C with surface-bound Gd from Type B, resulting in the highest Gd payload and broader labeling of neural stem/progenitor cells in the subventricular zone (SVZ) and neurons in the striatum.

These results underscore the importance of rational nanoparticle design to optimize cellular imaging. The presence of ssDNA is crucial for effective cellular uptake, while increased Gd loading enhances MR sensitivity. Type D GdAuNPs represent a promising platform for noninvasive labeling and detection of both neural progenitors and mature neurons using MRI and CT, whilst affording histological validation.

### 4.1. GdAuNP synthesis and characterization

The physicochemical properties of contrast agents, such as GdAuNPs, play a critical role in determining their interactions with biological systems, including the extracellular matrix and cellular uptake mechanisms [11,33]. For effective cellular imaging, contrast agents must not only be internalized by target cells but also retain sufficient T_1_ relaxivity to enhance MRI signal intensity [20]. T_1_-weighted imaging is particularly advantageous, as the resulting signal enhancement can be more confidently attributed to exogenous contrast agents, unlike susceptibility-based contrast that may arise from endogenous metals such as iron.

Although monomeric Gd chelates (e.g., Gd-DOTA) are widely used, their conjugation to gold nanoparticles significantly increases relaxivity due to enhanced local concentration and molecular packaging [34]. Incorporating both Gd and Au into a single nanoconstruct enables multimodal imaging via MRI and CT [35,36], while the addition of a fluorescent moiety facilitates independent verification by fluorescence microscopy. However, increasing the structural complexity of such agents necessitates stringent control over synthesis and characterization [29].

To systematically evaluate how design complexity influences *in situ* labeling and imaging performance, we synthesized four distinct GdAuNP formulations using single-stranded DNA (ssDNA) and two modes of Gd conjugation. Each synthetic step was validated using UV–visible spectroscopy, HPLC, and mass spectrometry, confirming successful ssDNA attachment, consistent Gd loading, and reproducible nanoparticle properties. The GdAuNPs exhibited narrow spectral features and minimal batch-to-batch variation in hydrodynamic diameter (~12 nm ± 1 nm), consistent with ssDNA functionalization.

Reproducibility was confirmed across multiple batches, demonstrating the robustness of the conjugation protocol and overall synthetic workflow. These results meet quality-by-design (QbD) standards for consistent nanoparticle production [37]. Long-term stability studies revealed that the particles retained their structural integrity for at least one year, with Type B GdAuNPs remaining stable for over three years—underscoring their suitability for long-term storage and delayed application. However, a greater understanding of the long-term stability within cells is desirable. Specifically, it remains unclear if ssDNA is degraded *in vivo* within cells and how this would affect its intracellular retention. Although AuNP are generally considered chemically and biologically inert, there is evidence that some biodegradation of these occurs intracellularly [38]. Further long-term physico-chemical and biological characterization of these GdAuNP in brain cells is hence required to more fully establish their potential for biological applications.

Sustained relaxivity over time is attributed to the high thermodynamic stability of the DOTA chelator, which minimizes Gd ion release—a key factor for both safety and imaging efficacy [39]. Because Gd-based agents can become sequestered within lysosomes after cellular uptake, resulting in signal quenching [13,15], the use of ssDNA offers an additional benefit. ssDNA promotes peri-nuclear localization and reduces lysosomal trafficking. Importantly, the random sequence of the ssDNA prevents genomic integration, while maintaining the GdAuNPs in a favorable intracellular location for preserving T_1_-weighted signal contrast [16]. Moreover, the use of DNA-based spacers allows spatial control over Gd-chelate positioning, which is known to influence MR relaxivity [40].

Among the four formulations, Type D GdAuNPs—featuring both ssDNA-conjugated and surface-conjugated Gd—exhibited the highest relaxivity and T_1_-weighted signal, highlighting the importance of maximizing Gd payload per particle. This design enabled detection of labeled cells post-implantation, as previously demonstrated with NSCs [16].

While spherical AuNPs were employed in this study due to their favorable tissue penetration, alternative geometries such as nanostars [41], nanodiamonds [42] and concave nanocubes [43] may offer further enhancements in relaxivity [44]. Multilayered architectures, including silica core/Au shell constructs, have also demonstrated improved *in vitro* relaxivity [45]. Although gold may contribute minimally to the particles’ magnetic profile [41,42], its effect is negligible relative to the dominant paramagnetic influence of Gd.

In summary, optimization of key design parameters—including nanoparticle geometry, size, surface chemistry, and Gd loading—is essential for enhancing the performance of GdAuNPs for cellular MRI [32,44]. Such advancements may ultimately enable cellular-resolution imaging, allowing for the precise anatomical localization of individual cells within complex tissue environments [46].

### 4.2. Impact of GdAuNP design on MRI and CT detection

The relaxivity of contrast agents is influenced not only by their physicochemical design but also by their biological microenvironment and access to free water [47]. In the brain, GdAuNPs may reside in aqueous environments, such as cerebrospinal fluid (CSF) in the ventricular system, or within tissue compartments, both extracellular and intracellular. Type C GdAuNPs exhibited greater T_1_-weighted (T1w) contrast in tissue than in the ventricles, while Type D GdAuNPs achieved comparable contrast across both environments. Although the ventricles offer unrestricted water access, they also dilute contrast agents significantly. Thus, constructs with lower Gd loading are more susceptible to dilution-related loss of signal. In tissue, signal dilution is determined more by the extent of nanoparticles permeating through the tissue. Type A GdAuNPs, which lack ssDNA, revealed a wider tissue distribution but achieved weaker contrast. In contrast, ssDNA-functionalized GdAuNPs were preferentially taken-up by cells, leading to less distribution with an enhanced contrast due to intracellular retention [16].

A defining feature of GdAuNPs is their multimodal detectability via MRI and CT [25,48]. While T1w MRI contrast depends on multiple factors—including relaxivity and tissue microenvironment [47]—CT signal is primarily dictated by the electron density of the contrast material [49]. Both Gd and Au contribute to CT contrast [35]. All nanoparticle designs showed modest CT contrast in the ventricles, with Type A generating the weakest signal. Tissue CT contrast was greatest for Type C GdAuNPs, which exhibited the least dispersion and thus highest local concentration. Notably, CT contrast inversely correlated with nanoparticle distribution volume, following a logarithmic relationship. Overall, contrast-to-noise ratio (CNR) was higher in MRI than CT across all types, except for Type C. Although Type D had the highest Gd loading, its broader distribution led to a modestly reduced CNR. Histological and SEM-EDS analysis corroborated this wider distribution of Type D GdAuNPs.

Surface functionalization plays a critical role in dictating nanoparticle biodistribution by modulating interactions with host proteins and the formation of a protein corona [50]. ssDNA-functionalized GdAuNPs exhibited a “bio-identity” that facilitated active cellular uptake in contrast to Type A particles, which lacked such functionality [51, 52]. Various peptides and proteins are known to enhance nanoparticle uptake [53–56], but surface elements can also trigger aggregation through protein interactions, potentially limiting both tissue penetration and intracellular access [57]. Aggregation may induce cytotoxicity [58], but no such effects were observed here. There was no increase in apoptosis in GdAuNP containing cells compared to non-labeled cells, further highlighting the low *in vitro* cytotoxicity of these types of agents [16]. Type A GdAuNPs, due to their limited surface modification, were not retained during immunohistochemical processing, while all ssDNA-containing GdAuNPs demonstrated excellent cellular uptake in both the SVZ and striatum.

### 4.3. In situ neuronal lineage uptake of GdAuNP

Intracerebroventricular injection of MRI contrast agents can result in passive uptake by neural stem/progenitor cells in the subventricular zone (SVZ), enabling imaging of their migration [19,23]. Iron oxide particles induce strong T2 contrast [59], but suffer from blooming artifacts, complicating precise localization. A T1-based contrast approach, such as that offered by GdAuNPs, minimizes distortion and improves anatomical resolution [46].

Although Type A GdAuNPs dispersed broadly and masked anatomical detail, T_2_-weighted images remained unaffected. In contrast, ventricles were clearly defined following Type C and D administration. ssDNA functionalization enabled efficient uptake along the ventricular wall. Approximately 20 % of SOX2+ cells in the SVZ internalized ssDNA-GdAuNPs, which also penetrated ~2 mm into adjacent parenchyma and labeled some mature neurons. GdAuNPs localized predominantly in the perinuclear region but also extended throughout the cytoplasm, consistent with prior *in vitro* studies [16]. Ependymal cells lining the ventricle showed limited uptake, although anterior ependymal and choroid plexus cells were endocytosing GdAuNPs.

To assess *in situ* neuronal labeling, GdAuNPs were injected directly into striatal tissue. Effective labeling depends on contrast agent distribution, concentration, and surface chemistry [60]. Type A GdAuNPs showed poor neuronal uptake, particularly at distal sites from the injection site. Type C particles were most effective at labeling neurons, likely due to limited diffusion and elevated local concentration. Type D GdAuNPs also labeled neurons efficiently near the injection site and in peripheral regions. Type B showed weaker performance, potentially due to the absence of Gd chelates on the ssDNA. The selective neuronal uptake of ssDNA-GdAuNPs is notable, given the non-specificity of random DNA sequences. This compares favorably to methods requiring genetic modification (e.g., AAV-mediated expression of *oatp1a* for Gd-EOB-DTPA uptake [18]). Thus, ssDNA-functionalized GdAuNPs represent a simple yet effective platform for targeted neuronal imaging, although further mechanistic studies are needed to elucidate the basis of this apparent selectivity [52].

Beyond neurons, GdAuNPs were also detected in microglia, which were abundant in tissue. Few macrophages were observed, consistent with minimal injury-induced infiltration. Up to 50 % of microglia and macrophages contained GdAuNPs, though neurons exhibited the highest labeling efficiency. Intracellular localization differed between cell types. In microglia, GdAuNPs accumulated peri-nuclearly and in cellular processes, suggesting uptake from extracellular space followed by nuclear-directed transport. In contrast, macrophages sequestered the nanoparticles within cytoplasmic phagosomes, indicative of phagocytosis rather than endocytosis. The small size of GdAuNPs likely limited macrophage uptake, while favoring endocytic internalization in neurons and glia. No increased macrophage recruitment was observed in response to GdAuNPs. However, in pathological conditions with greater immune activation (e.g., traumatic brain injury), this cellular distribution of GdAuNPs may shift significantly [61].

## 5. Conclusions

GdAuNPs provide a modular and versatile platform for the development of multifunctional cellular imaging agents. In this study, we systematically evaluated how variations in surface chemistry influence the MRI sensitivity and cellular labeling efficiency of GdAuNPs within the subventricular zone (SVZ). Four distinct GdAuNP designs were synthesized and thoroughly characterized, each demonstrating high reproducibility, long-term stability (shelf life >1 year), and minimal batch-to-batch variability.

Functionalization with ssDNA proved critical for achieving effective *in situ* cellular uptake. Type A GdAuNPs, which lacked ssDNA, failed to robustly label cells *in vivo*. While both Type A and Type D constructs exhibited high *in vitro* relaxivity, Type D achieved the highest contrast-to-noise ratio *in vivo*.

Neuronal lineage cells efficiently internalized ssDNA-functionalized GdAuNPs, with labeling observed in over 80 % of the population and a characteristic perinuclear distribution. Microglia also internalized ssDNA-GdAuNPs, albeit with lower efficiency (~20 %), whereas macrophages exhibited phagocytic uptake of GdAuNPs, with signal confined to phagosomes.

Among all tested formulations, Type D—featuring both ssDNA-conjugated and surface-bound Gd—offered the most favorable balance of cellular uptake, Gd payload, and multimodal imaging performance. These agents hold strong potential for the non-invasive tracking of neural stem cells and other non-phagocytic cell types during development, regeneration, and disease progression.

## Supplementary Material

mmcgigs2

mmcgigs5

mmcgigs10

mmcgigs11

mmcgigs7

mmcgigs17

mmcgigs4

mmcgigs13

mmcgigs6

mmcfigs9

mmcfigs8

mmcfigs1

mmcfigs3

mmcfigs16

mmcfigs12

mmcfigs15

mmcfigs14

## Figures and Tables

**Table 1:** MR imaging parameters (TE – echo time; TR – repetition time; NA - number of averages; FOV – field of view).

Aim	Method		TE (ms)	TR (s)	NA	FOV (mm)	Time

Relaxometry	RARE-VTR	T_1_ map	25.24	0.1–15	2	25.6 × 12.8	18 min 30 s
	MSME	T_2_ map	20–600	5	2		21 min 20 s
	FLASH	T_1_w	4.41	0.05	8		1 min 17 s
3D imaging	FLASH	T_1_w	7	0.05	2	35.2 × 38.4 × 16	131 min 25 s
	RARE	T_2_w	40	1.8	1		31 min 41 s

**Table 2:** List of primary and secondary antibodies.

Antibody (host)	Clone	Fluorochrome	Dilution	Company	Cat. Ref.

SOX2 (rabbit)	SP76	n/a	1:1000	Abcam	Ab93689
NeuN (mouse)	1B7	n/a	1:1000	Abcam	Ab104224
CX3CR1 (rabbit)	EPR24267-2	n/a	1:1000	Abcam	Ab308613
CD68 (mouse)	ED1	n/a	1:1000	Abcam	Ab31630
Caspase-3 (rabbit)	polyclonal	n/a	1:200	Millipore	AB3623
Anti-rabbit (donkey)	polyclonal	Alexa Fluor 647	1:1000	Invitrogen	A31573
Anti-rabbit (donkey)	Polyclonal	Alexa Fluor 488	1:1000	Abcam	Ab150061
Anti-mouse (goat)	polyclonal	Alexa Fluor 488	1:1000	Invitrogen	A11001

**Table 3:** Polydispersity index (PDI) measurements for different GdAuNP formulations revealed monodisperse samples with no concentration-dependent aggregation.

PDI	Type A	Type B	Type C	Type D

10 nM	0.062	0.11	0.26	0.14
100 nM	0.070	0.18	0.25	0.14

**Table 4:** 1.41T relaxivity (mM^−1^s^−1^) measurements for Type A, B, C, D GdAuNPs (37 °C).

1.41T	r_1_-ionic	r_1_-molecular	r_2_-ionic	r_2_-molecular

Type A	23.19	22828	53.75	52906
Type B	19.04	11502	44.36	26796
Type C	24.04	4077	64.40	10923
Type D	22.28	20666	50.12	46500

9.4T	r_1_-ionic	r_1_-molecular	r_2_-ionic	r_2_-molecular

Type A	3.403	3349	44.56	43860
Type B	4.450	2465	62.86	32350
Type C	5.603	950	90.29	15313
Type D	4.244	3937	49.14	45591

**Table 5:** Summary of physicochemical characteristics of contrast agents.

Measure		ProHance	GdAuNP			
	
			Type A	Type B	Type C	Type D

Size (nm)		n/a	13	12.6	13	13
[Gd] (μM)		5 × 10^5^*	492	551	85	464
[AuNP] (nM)		n/a	500	964	500	500
Gd/AuNP		n/a	984	572	170	928
DNA strands/AuNP		n/a	n/a	73	39	60
[Gd]	r_1_ (mM^−1^s^−1^)	5.165	3.464	3.716	5.934	3.742
	r_2_ (mM^−1^s^−1^)	5.349	47.53	56.71	106	45.85
[AuNP]	r_1_ (mM^−1^s^−1^)	n/a	3409	2448	1006	3168
	r_2_ (mM^−1^s^−1^)	n/a	46780	34330	17980	38820

Relaxivity measurements were performed at 9.4T.

* Concentration of ProHance

## Data Availability

Data will be made available on request.

## Appendix A. Supplementary data

Supplementary data to this article can be found online at https://doi.org/10.1016/j.biomaterials.2025.123947.
