# Supplementary figures and images for: Design and characterization of in situ cell-penetrating multi-modal gadolinium-gold nanoparticles for MR and CT imaging
Source: Biomaterials. Author manuscript; Available in PMC 2026 Aug 4. (PMC13435245; doi:10.1016/j.biomaterials.2025.123947)

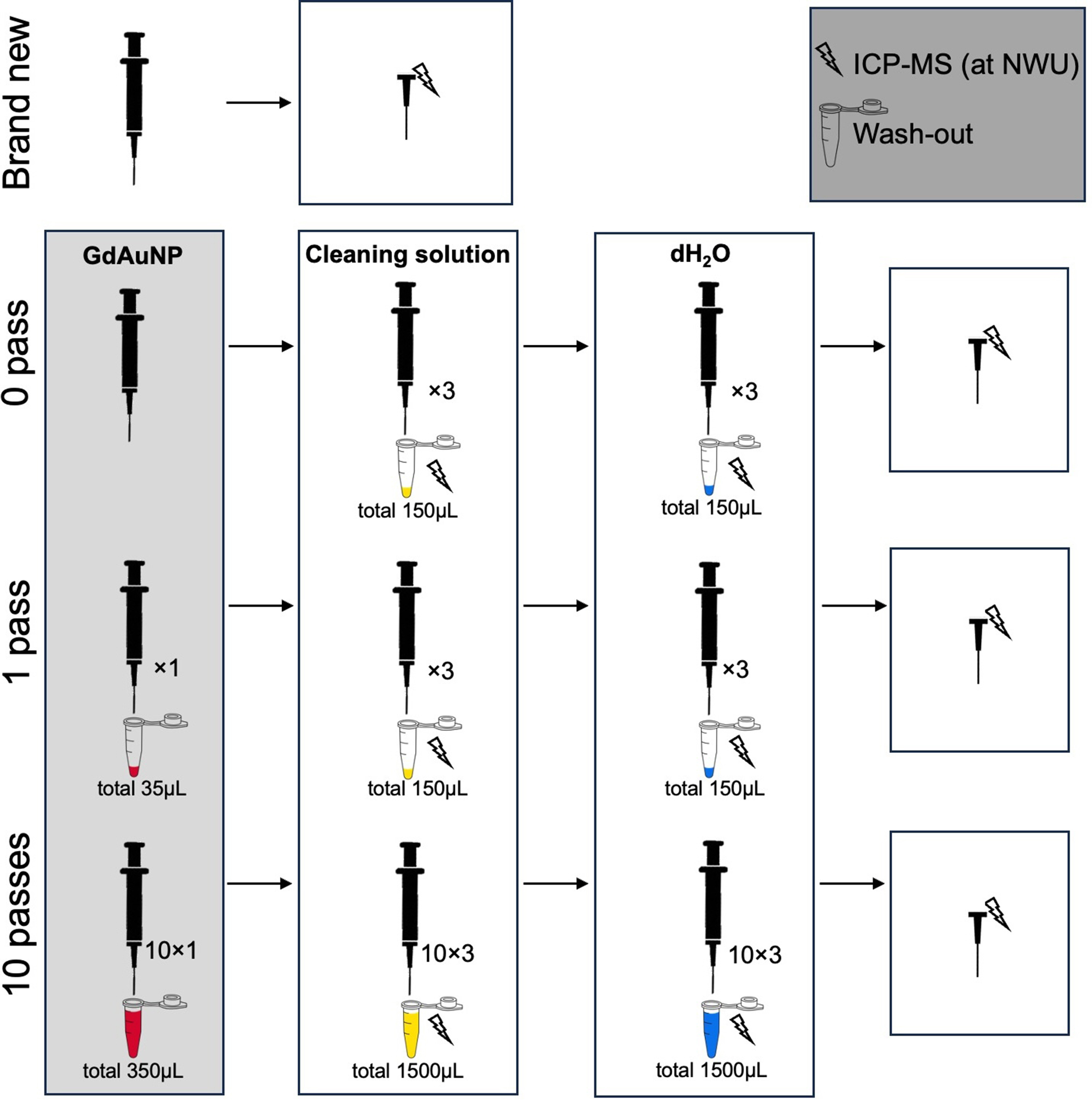

Supplement: mmcgigs2 [file NIHMS2189822-supplement-mmcgigs2.jpg]

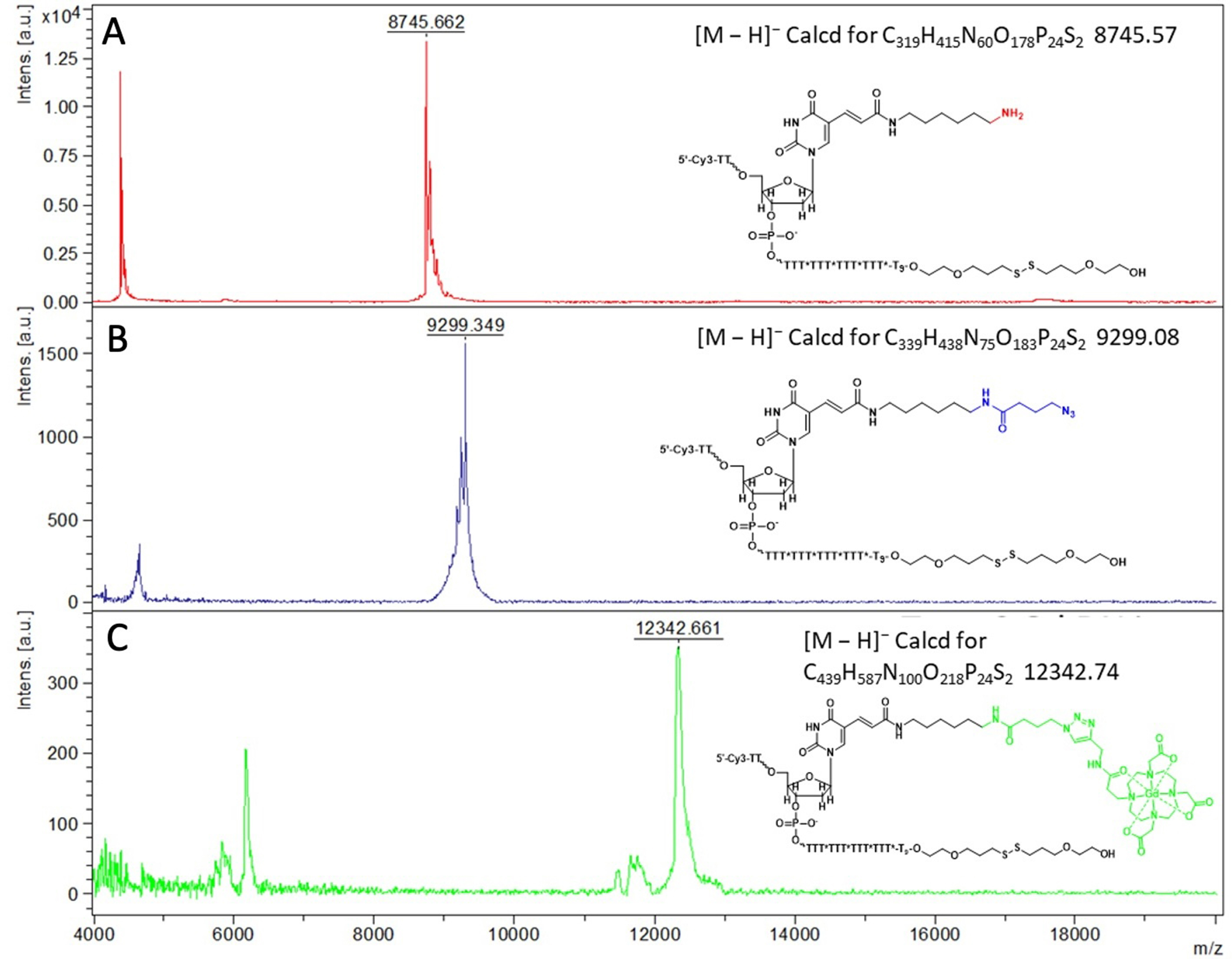

Supplement: mmcgigs5 [file NIHMS2189822-supplement-mmcgigs5.jpg]

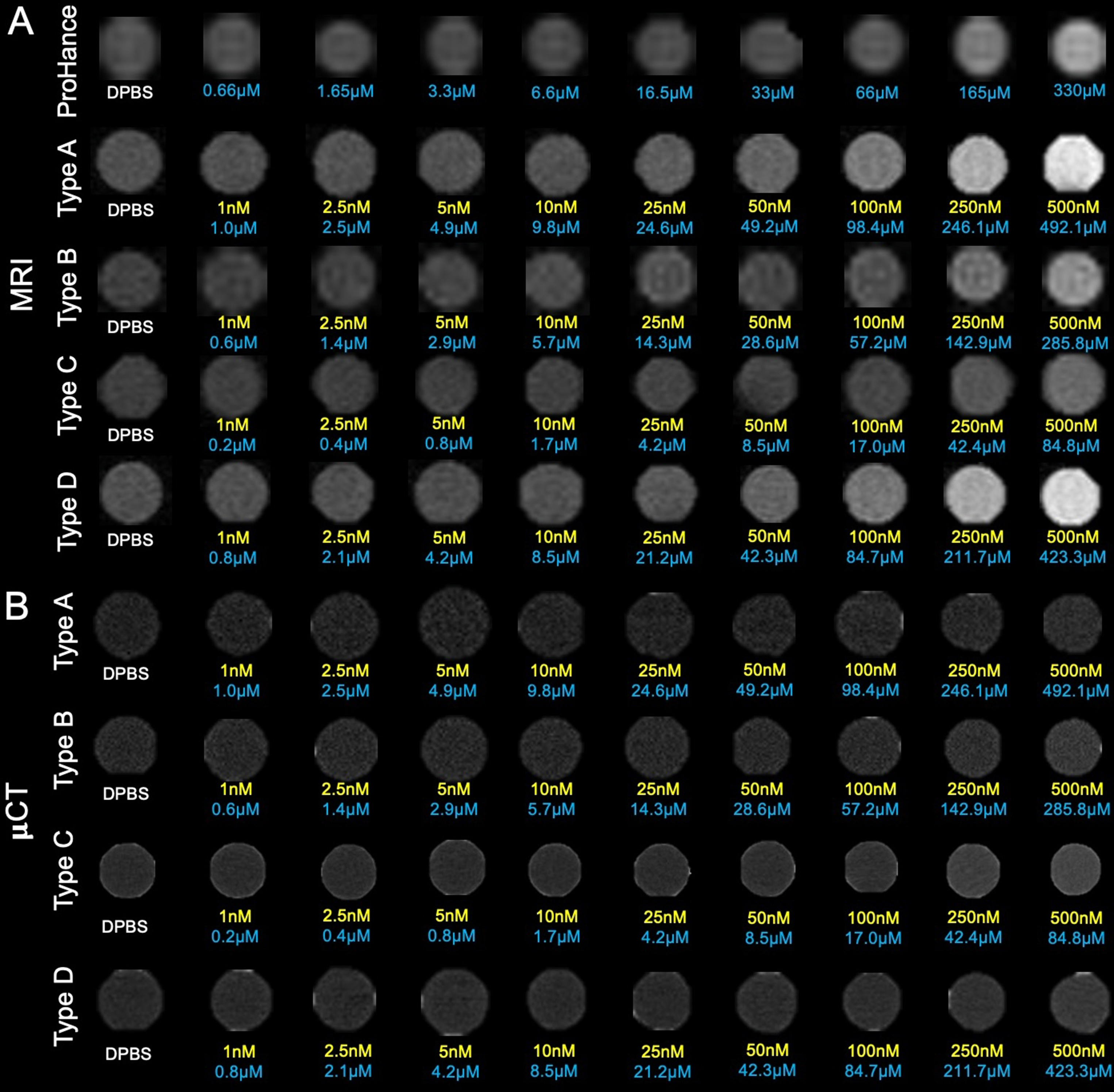

Supplement: mmcgigs10 [file NIHMS2189822-supplement-mmcgigs10.jpg]

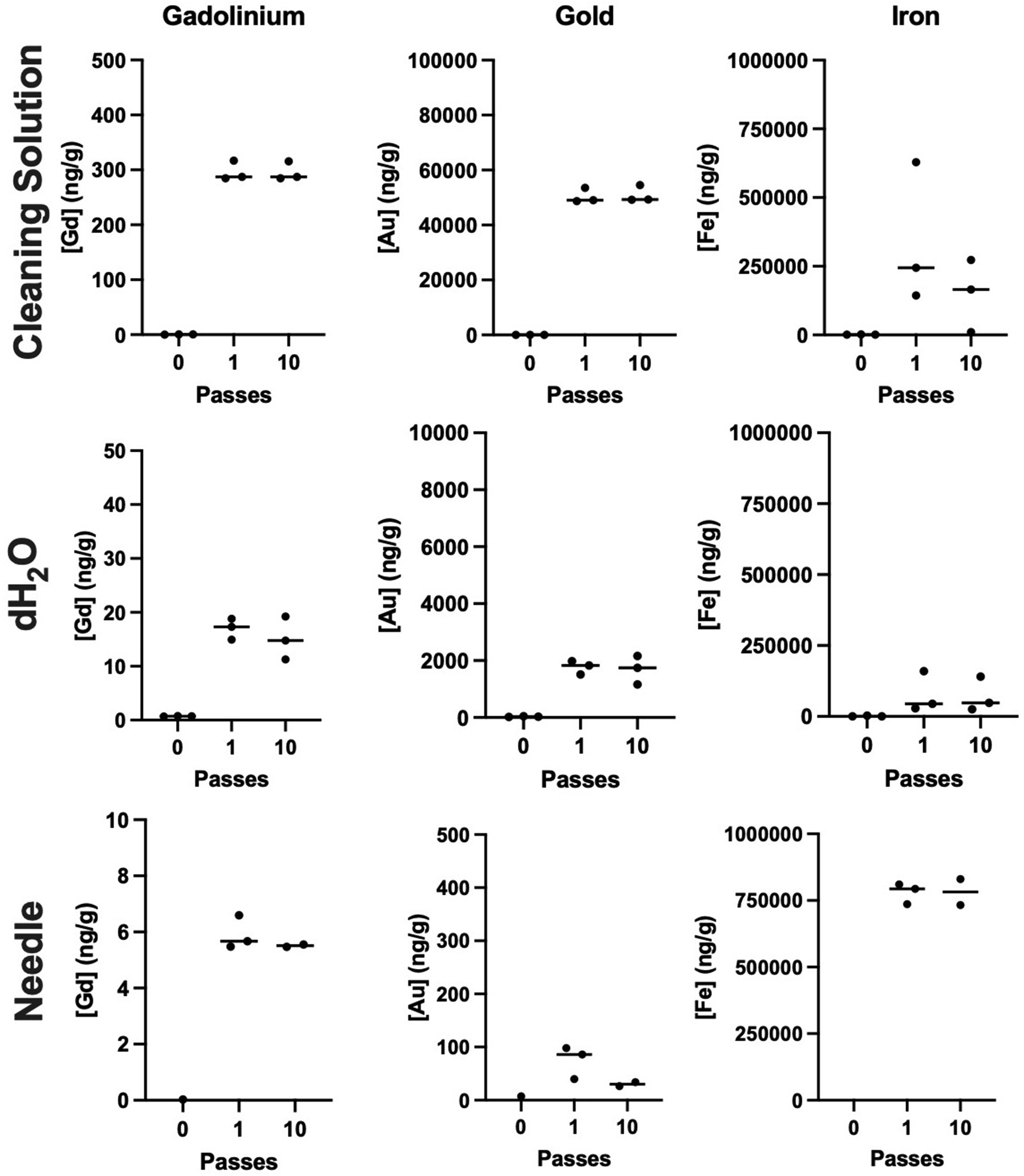

Supplement: mmcgigs11 [file NIHMS2189822-supplement-mmcgigs11.jpg]

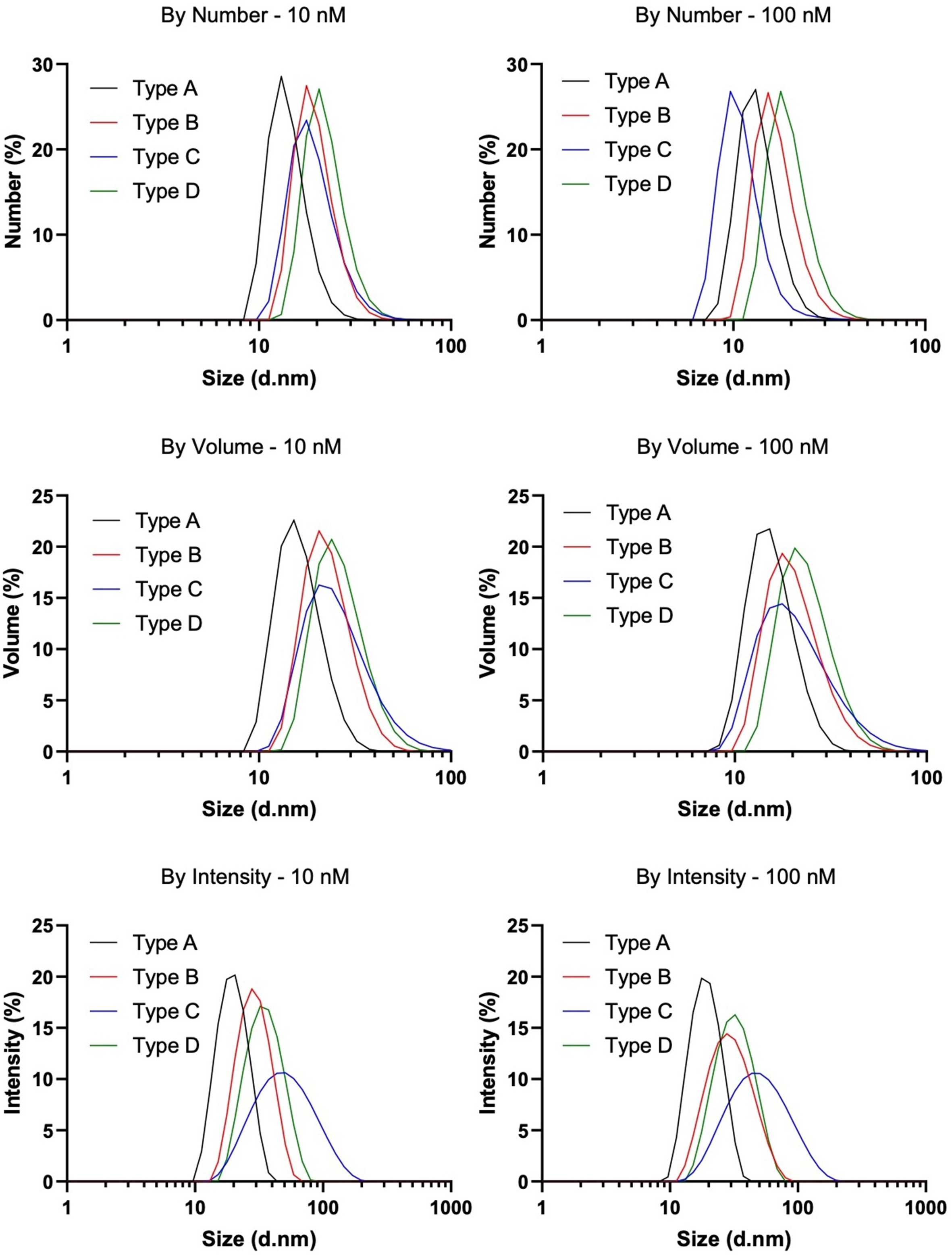

Supplement: mmcgigs7 [file NIHMS2189822-supplement-mmcgigs7.jpg]

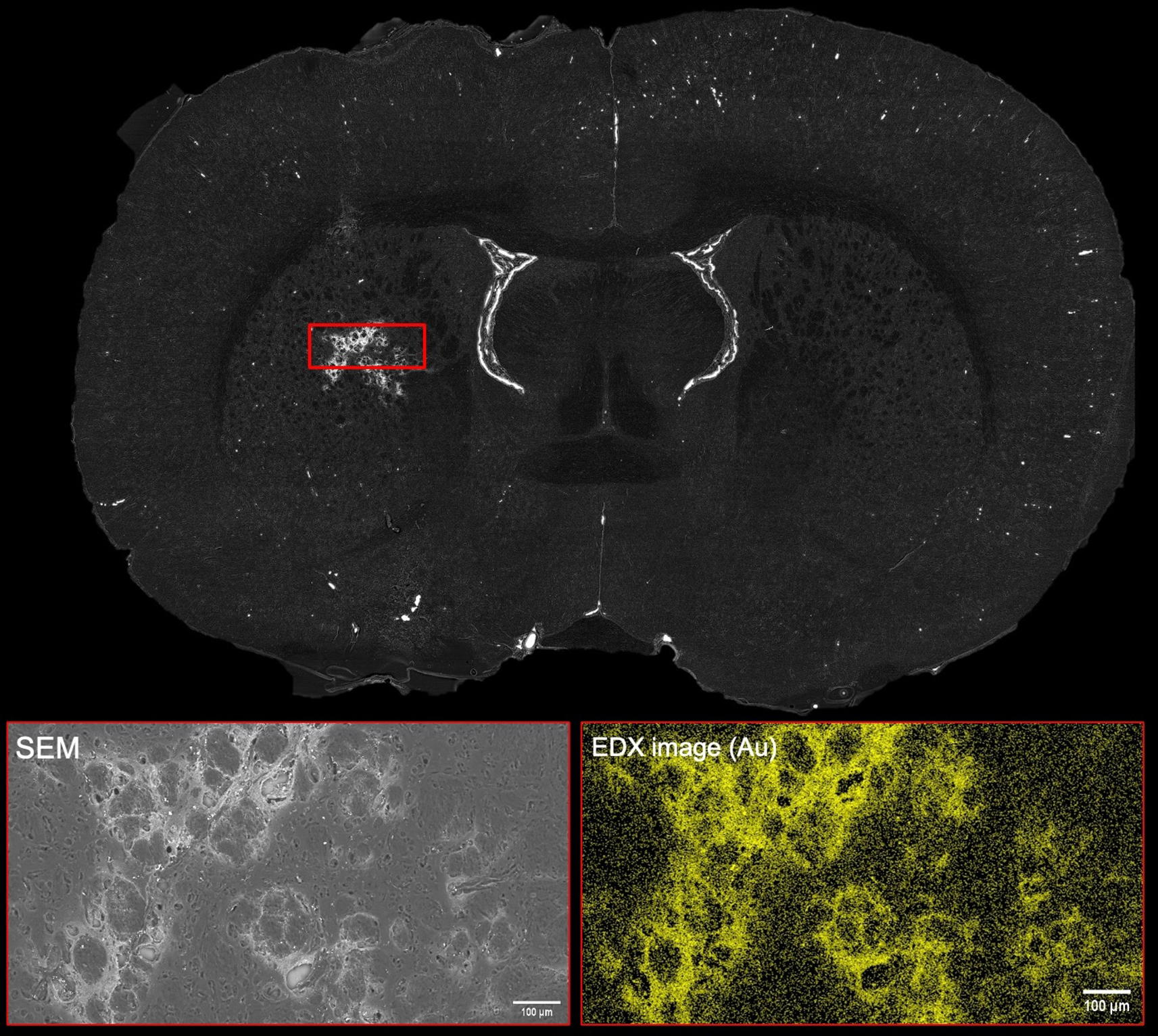

Supplement: mmcgigs17 [file NIHMS2189822-supplement-mmcgigs17.jpg]

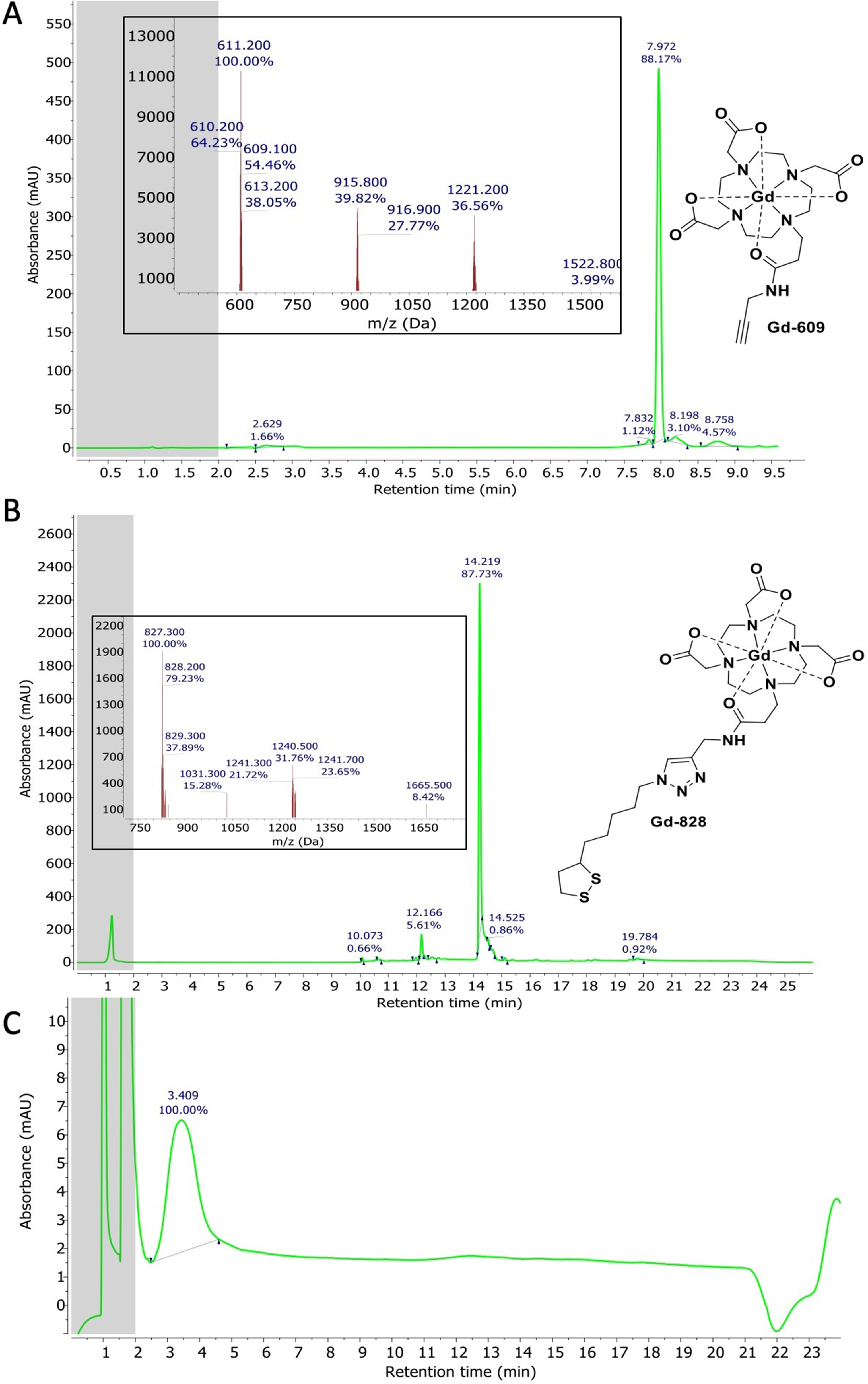

Supplement: mmcgigs4 [file NIHMS2189822-supplement-mmcgigs4.jpg]

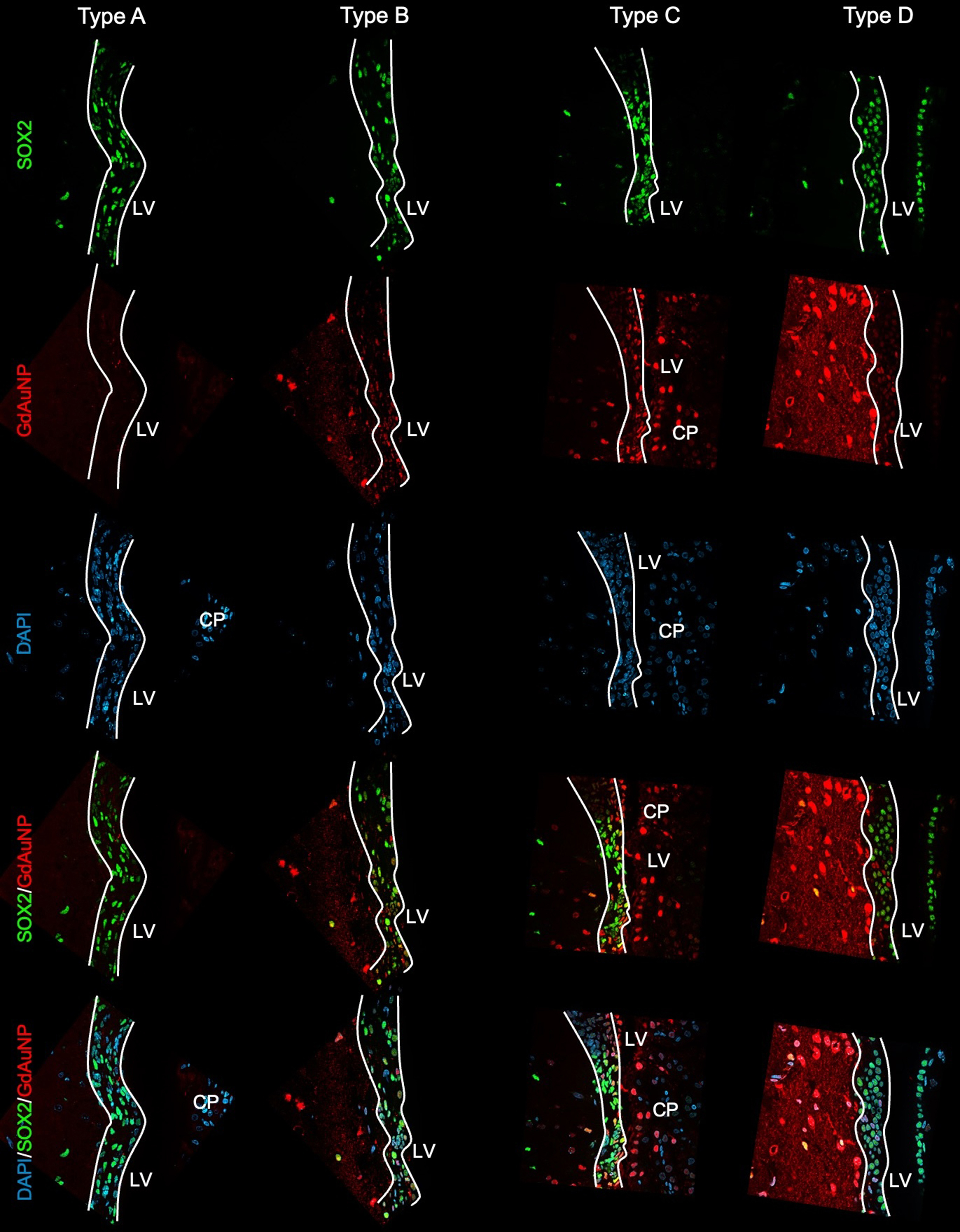

Supplement: mmcgigs13 [file NIHMS2189822-supplement-mmcgigs13.jpg]

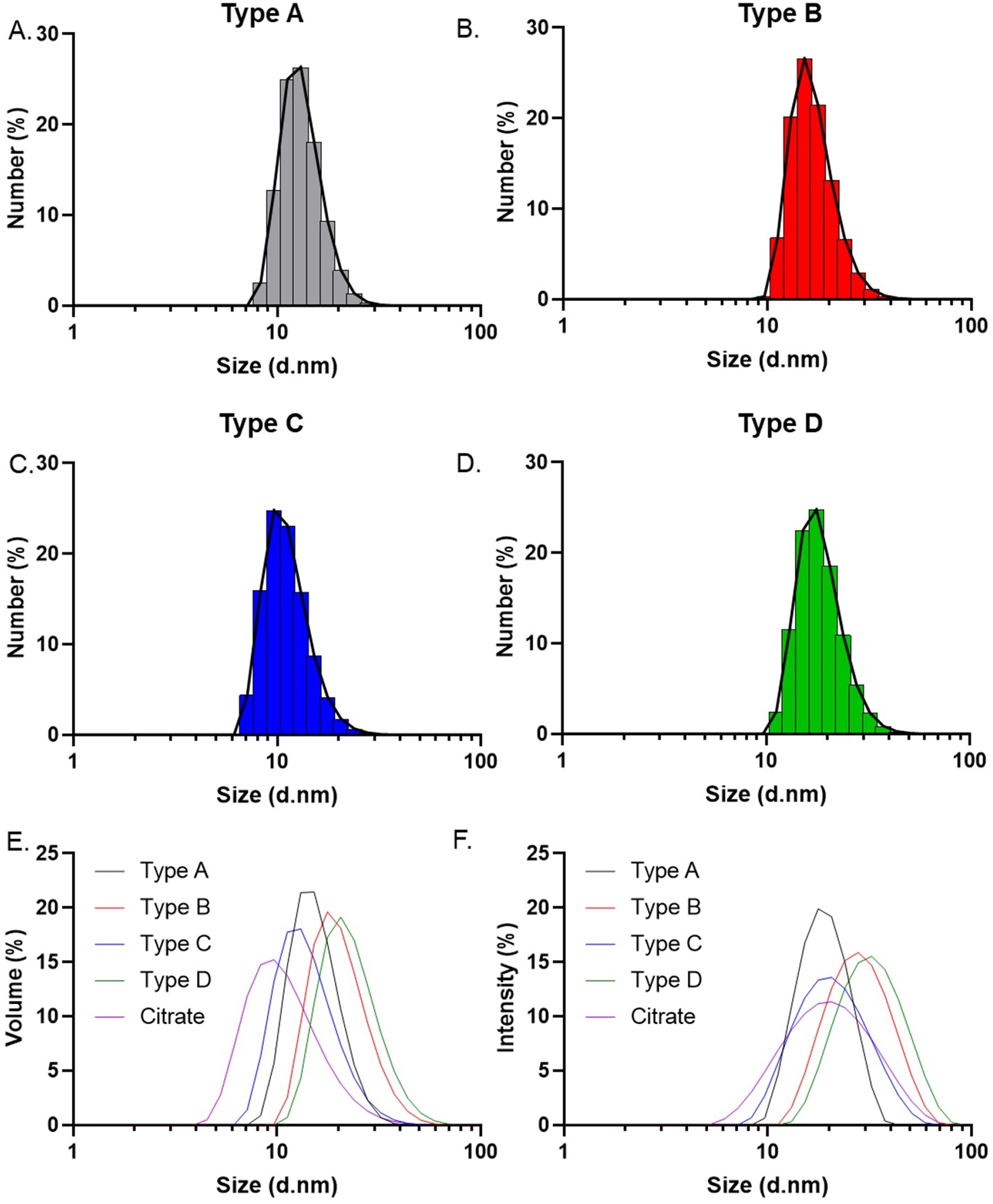

Supplement: mmcgigs6 [file NIHMS2189822-supplement-mmcgigs6.jpg]

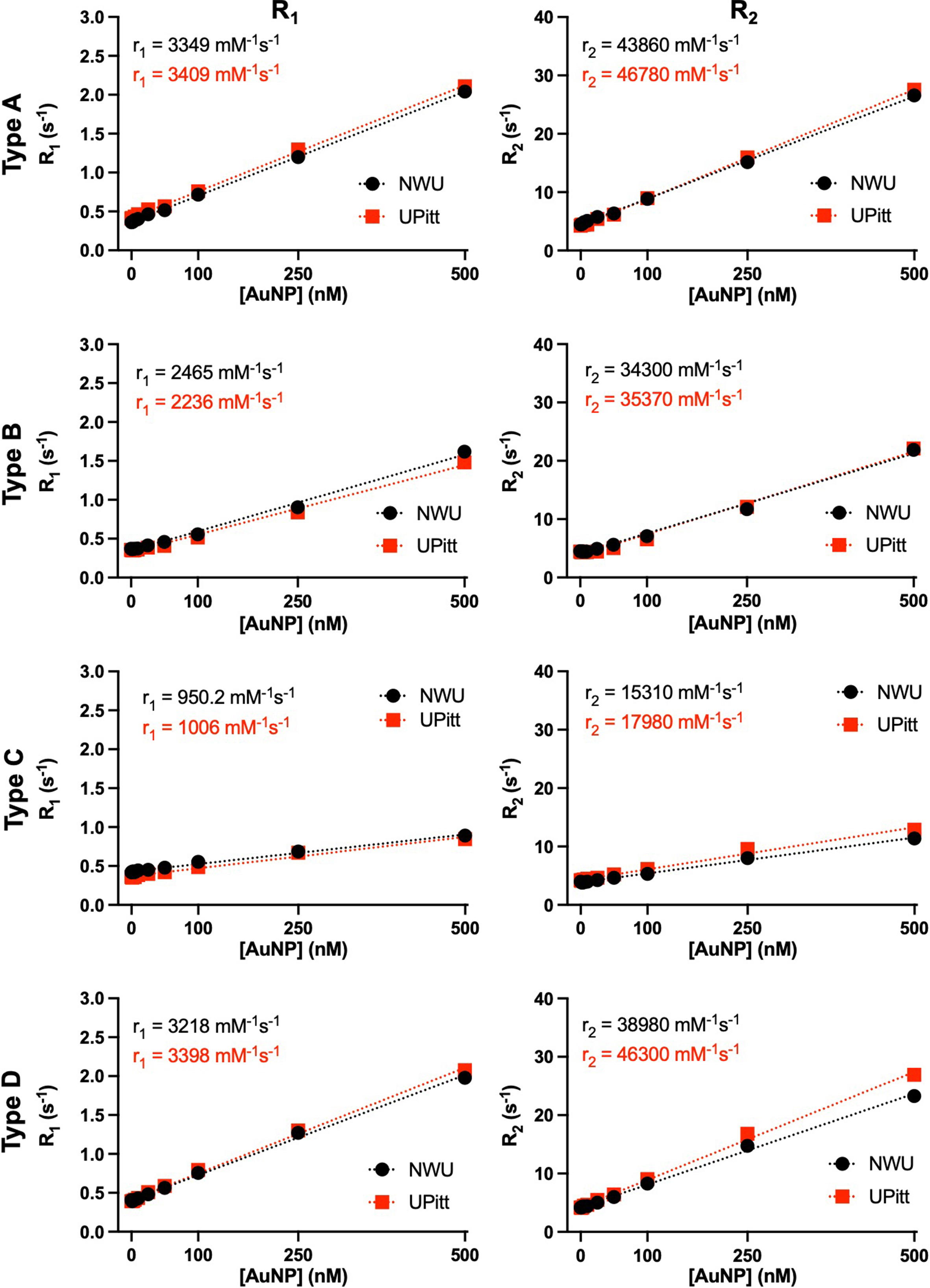

Supplement: mmcfigs9 [file NIHMS2189822-supplement-mmcfigs9.jpg]

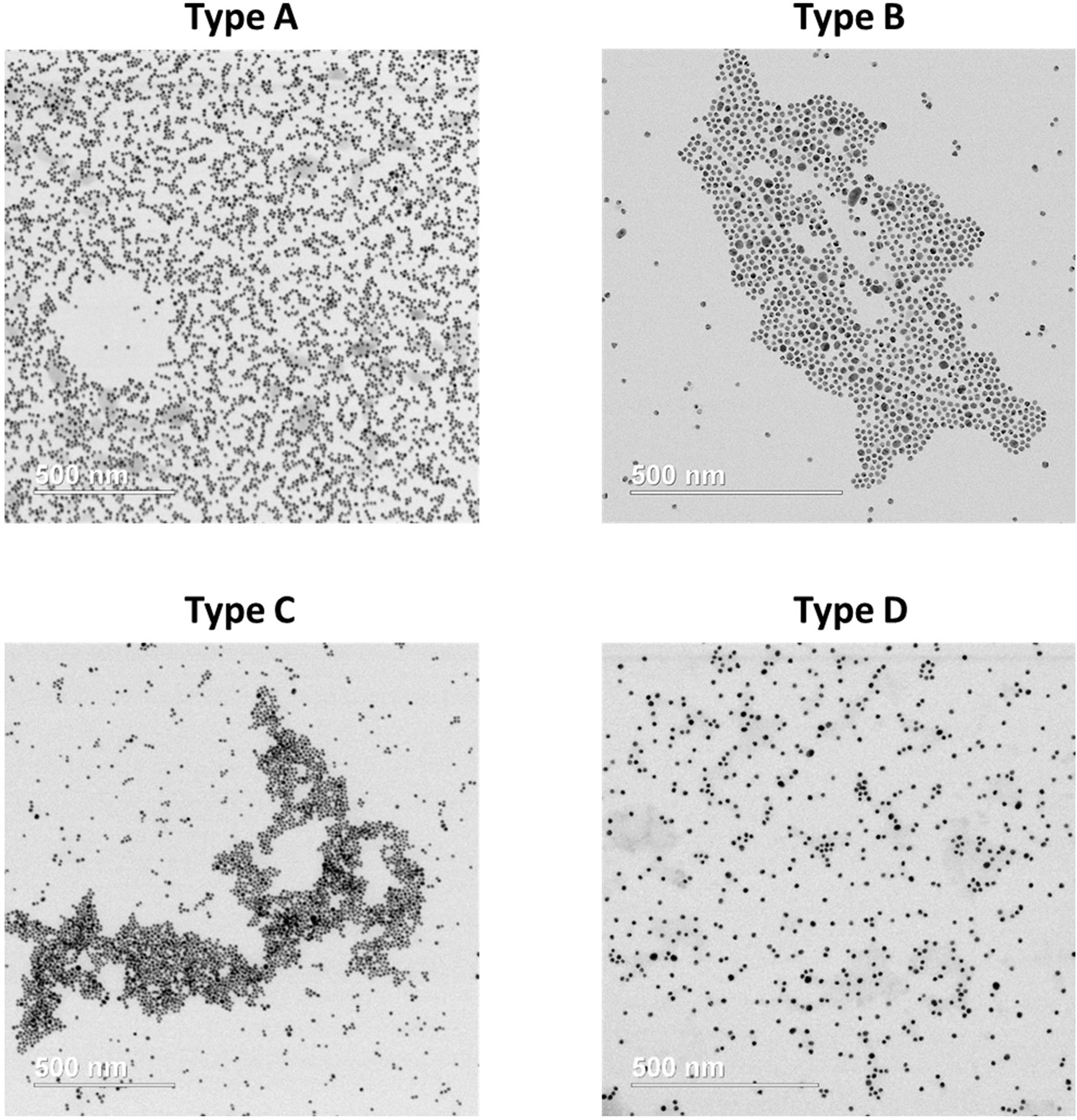

Supplement: mmcfigs8 [file NIHMS2189822-supplement-mmcfigs8.jpg]

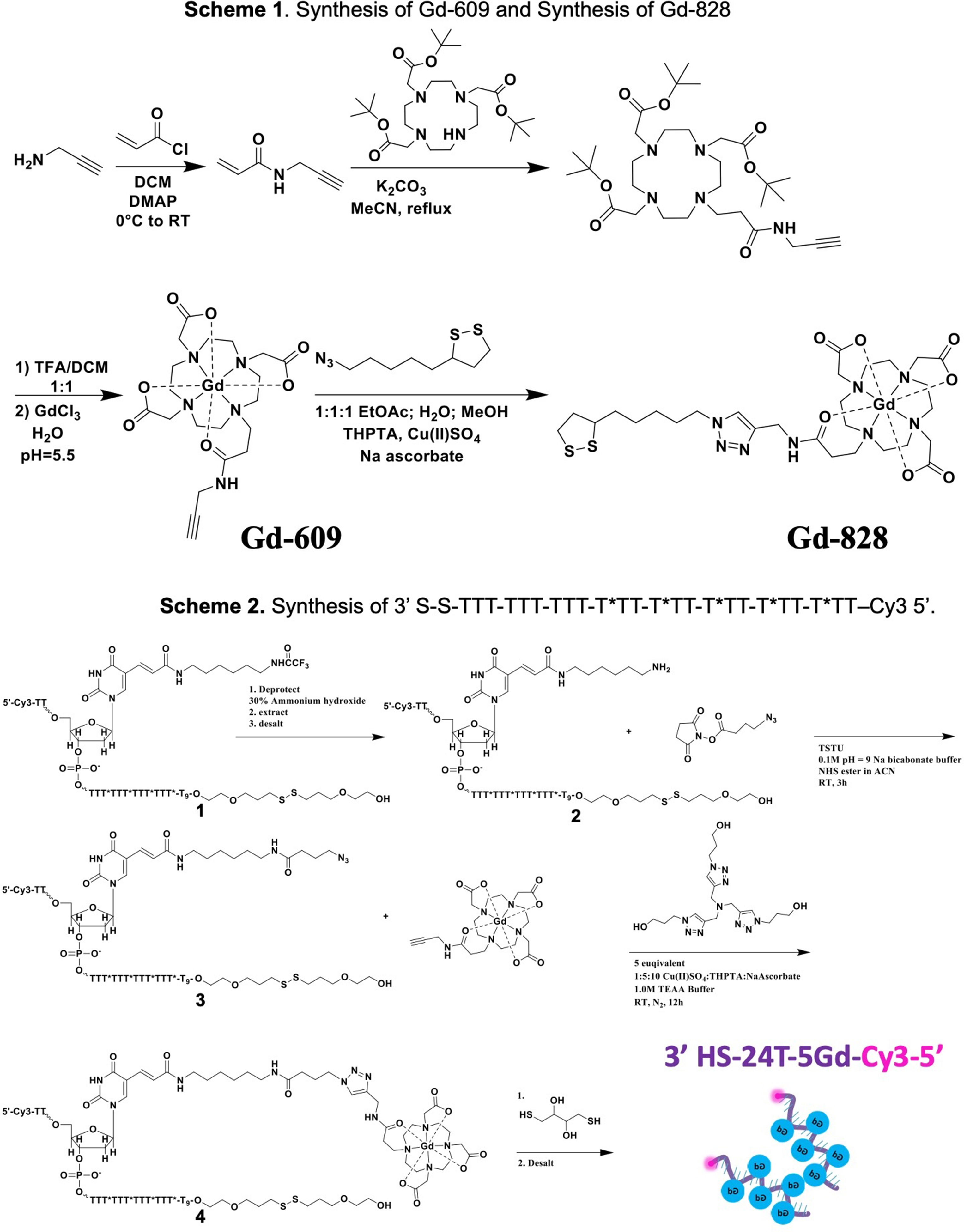

Supplement: mmcfigs1 [file NIHMS2189822-supplement-mmcfigs1.jpg]

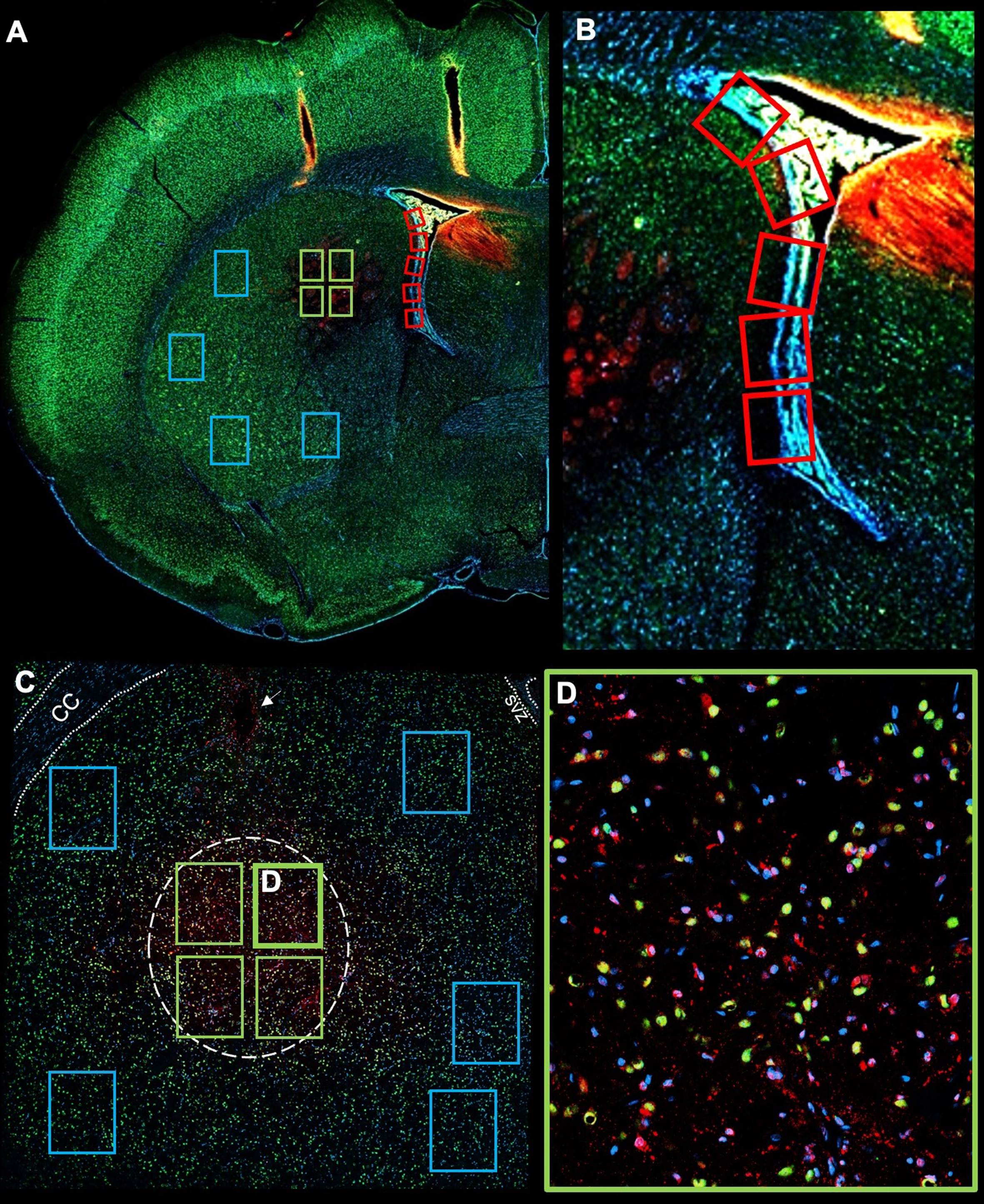

Supplement: mmcfigs3 [file NIHMS2189822-supplement-mmcfigs3.jpg]

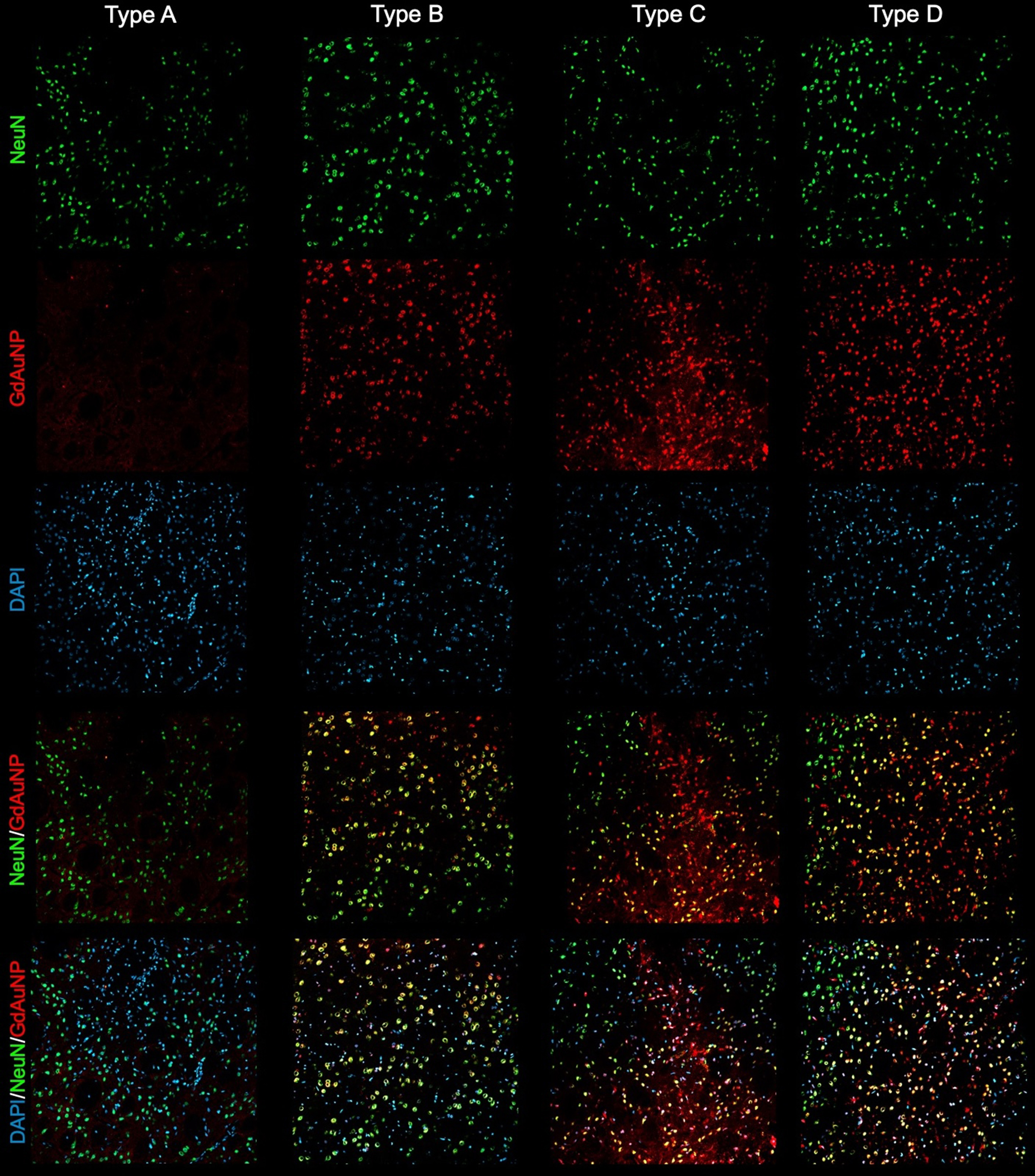

Supplement: mmcfigs16 [file NIHMS2189822-supplement-mmcfigs16.jpg]

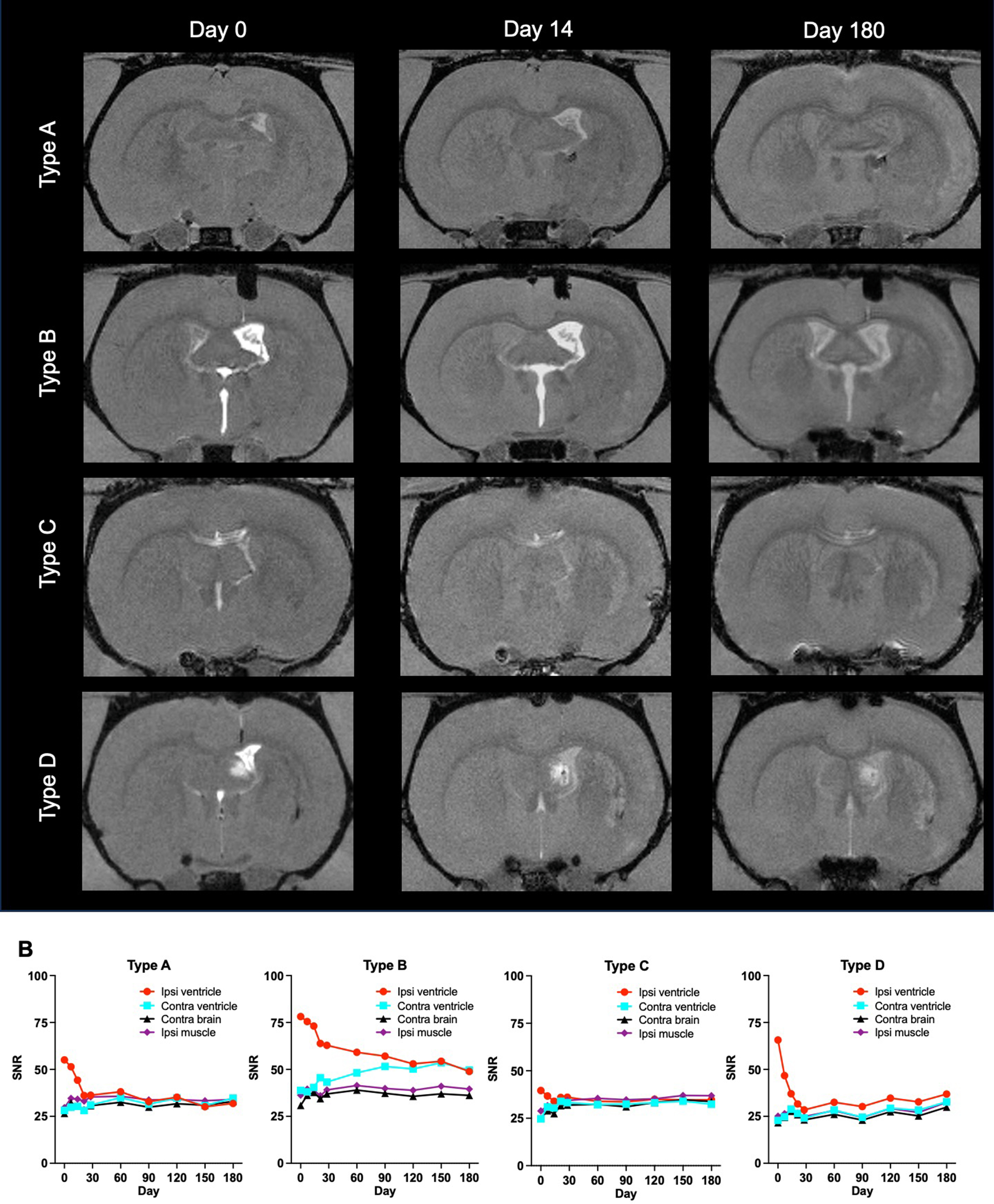

Supplement: mmcfigs12 [file NIHMS2189822-supplement-mmcfigs12.jpg]

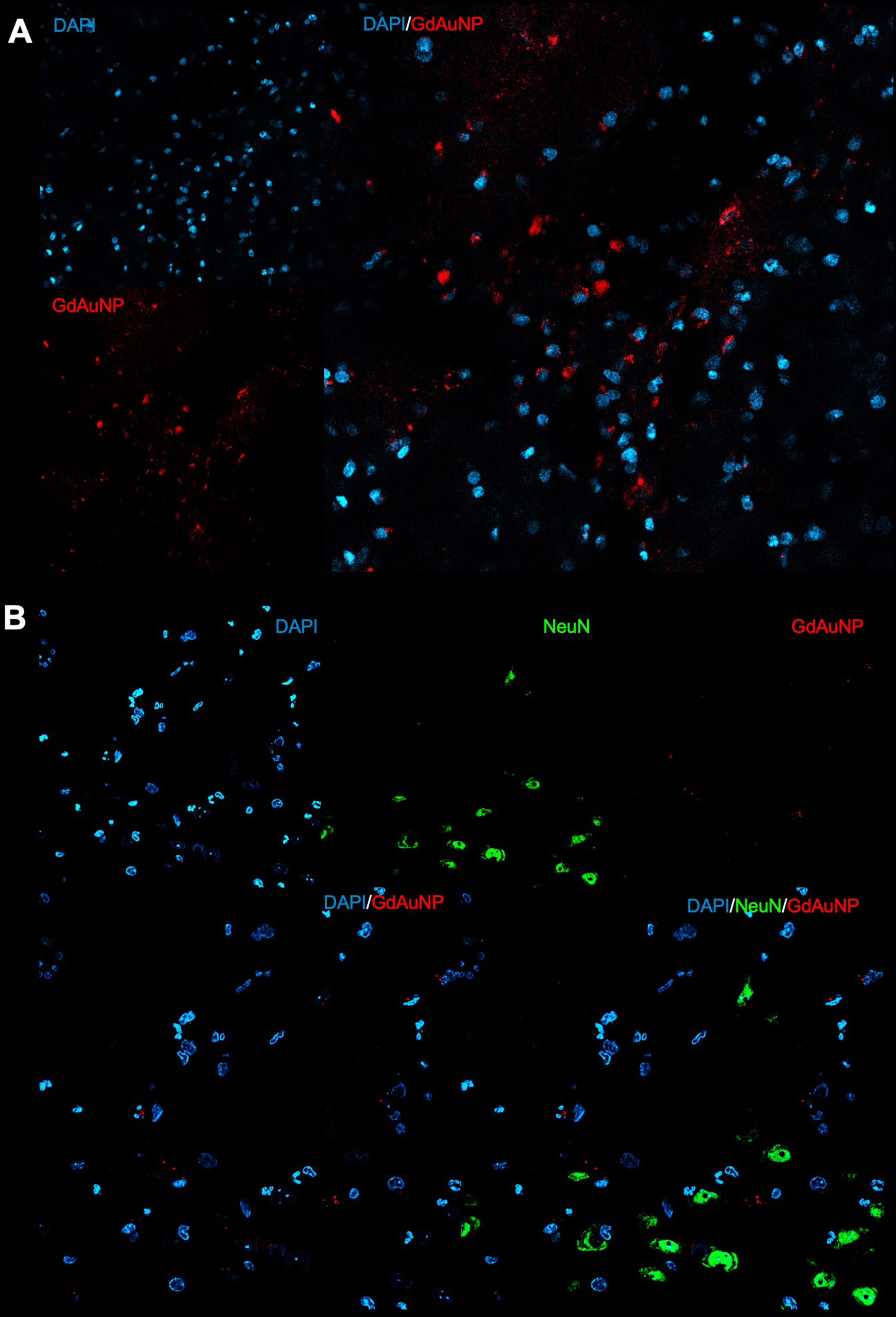

Supplement: mmcfigs15 [file NIHMS2189822-supplement-mmcfigs15.jpg]

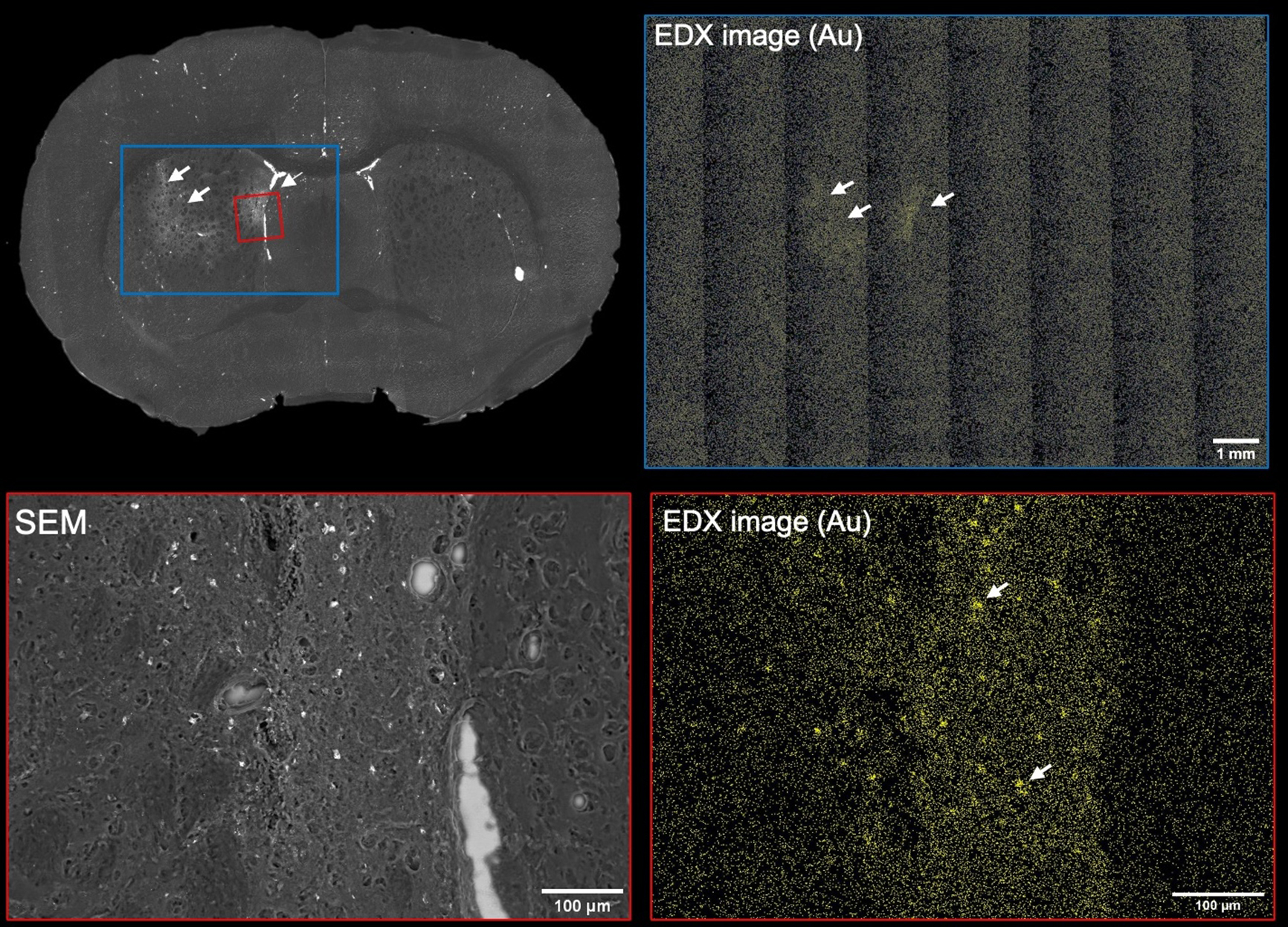

Supplement: mmcfigs14 [file NIHMS2189822-supplement-mmcfigs14.jpg]
